# Triplet Electron Exchange in Carbon Nanodots‐assisted Long‐persistent near‐infrared Chemiluminescence for Oncology Synergistic Imaging and Therapy

**DOI:** 10.1002/advs.202411898

**Published:** 2024-12-11

**Authors:** Run‐Wei Song, Tian‐Ci Jiang, Xue‐Yang Zhang, Cheng‐Long Shen, Qing Lou, Chong‐Xin Shan

**Affiliations:** ^1^ Henan Key Laboratory of Diamond Optoelectronic Materials and Devices Key Laboratory of Material Physics Ministry of Education and School of Physics and Laboratory of Zhongyuan Light Zhengzhou University Zhengzhou 450052 China; ^2^ Department of Respiratory and Critical Care Medicine The First Affiliated Hospital of Zhengzhou University Zhengzhou 450052 China; ^3^ College of Public Health Zhengzhou University Zhengzhou 450052 China

**Keywords:** biological imaging, carbon nanodots, chemiluminescence, photodynamic therapy, triplet electron exchange

## Abstract

In classical photodynamic therapy, tumor cells are killed by the cytotoxic species via type‐I/II photochemical reactions, seriously limited by the external photoexcitation and hypoxia. Herein, the electron transfer mechanism between fluorophores and peroxalate‐H_2_O_2_ reaction is investigated and the singlet/triplet electron exchange is utilized to achieve long‐persistent chemiluminescence imaging and synergistic type‐I/II/III photodynamic therapy. As a proof‐of‐concept, the photosensitizers of carbon nanodots (CDs)‐loaded chlorin e6 (CDs‐Ce6) are designed and integrated with the peroxalate molecules, and the as‐prepare polymer carbon nanodots (*p*‐CDs) exhibit novel tumor microenvironment (TME)‐responsive long‐persistent near‐infrared CL and photochemical reactions, evoking the in vivo imaging and synergistic dynamic therapy in tumor tissue. Mechanistically, the excess reactive oxygen species in TME can trigger the chemically initiated singlet/triplet electron exchange between the hydrophobic CDs‐Ce6 and peroxalate‐derived 1,2‐dioxetanes and thus the excess excited singlet/triplet electron of the CDs‐Ce6 can ensure the long‐persistent near‐infrared CL, type I/II photochemical production of hydroxyl radicals, superoxide radical and singlet oxygen, and type III photochemical damage of maladjusted biomacromolecules, enabling the long‐persistent near‐infrared biological imaging and enhanced cancer therapy. These results shed a new sight into the energy transfer mechanism in chemiluminescence and pave a new sight into the architecture of multifunctional theragnostic nanoplatforms.

## Graphical abstract

The CDs‐loaded chlorin e6 (CDs‐Ce6) are designed and integrated with the peroxalate molecules, and thus the excess reactive oxygen species in tumor microenvironment can trigger the chemically initiated electron exchange between hydrophobic CDs‐Ce6 and peroxalate‐derived 1,2‐dioxetanes, evoking the excess excited singlet/triplet electron for a long‐persistent near‐infrared CL emission, type I/II photochemical production, and type III photochemical damage of maladjusted biomacromolecules.

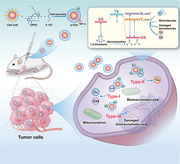

## 1 Introduction

Involving the administration of light‐activated photosensitizers (PSs) and the excess generation of cytotoxic hydroxyl radicals (·OH), superoxide radical (·O_2_^–^) and singlet oxygen (^1^O_2_) to induce cell apoptosis, the photodynamic therapy (PDT) has gained tremendous attention in various diseases, especial for the precise cancer therapy.^[^ ^1^ ^]^ Compared with the traditional chemotherapy, radiotherapy, and surgery, diverse advantages of the PDT for cancer therapy can be concluded, including minimal invasive, negligible drug resistance, and controllable selection of treatment region.^[^ ^2^ ^]^ However, due to the inherent disadvantages of external photoexcitation, autofluorescence and photobleaching, there are various limitations of classical PDT in solid tumor or deep‐tissue tumors.^[^ ^3^ ^]^ Recently, there are numerous researches on the PDT strategies assisted by endogenous self‐illumination. On the one hand, with the novel interior environment, featuring as the high levels of metabolism, maladjusted biosynthesis intermediates, acidosis, and hypoxia, etc., in tumor, the tumor microenvironment (TME)‐responsive photoluminescence (PL), bioluminescence (BL), or chemiluminescence (CL) can realize a spatiotemporal resolution optical imaging, and thus provide an excellent approach to the quantification and localization of tumor tissues, enabling the precise diagnosis and therapy of cancers.^[^ ^4^ ^]^ On the other hand, the inflammatory‐responsive in vivo BL or CL emission can be further utilized as endogenous illumination to excite PSs and produce various cytotoxic species, enabling the TME‐responsive in vivo synergistic biological imaging and therapy simultaneously.^[^ ^3^, ^5^ ^]^ With the main responsibility for tumor progression, aggressive metastasis and high drug resistance, these TME‐responsive combinations of biological imaging and dynamic therapy have drawn extreme attentions on the development of anticancer strategies with effective therapeutic targets specifically toward carcinoma cells.^[^ ^2^, ^4^, ^6^ ^]^ To date, the employment of intelligent materials with TME‐responsive theragnostic capacities has been proposed as one of the most ideal ways, which exhibits obvious advantages to enhance therapeutic efficiency with minimized side effects.

Generally, high level of reactive oxygen species (ROS) derived from the oxygen metabolism in tumor cells have been extensively approved to be reactive and signaling molecules, endowing various intelligent materials with the capability of TME‐responsive in vivo biological imaging and therapy.^[^ ^4^, ^7^ ^]^ With the inflammatory products (e.g., hydrogen peroxide, H_2_O_2_) as the reactant, the long‐persistent long‐wavelength CL emission from the fluorophores and peroxalate‐H_2_O_2_ or luminol‐H_2_O_2_ reaction has been demonstrated as an ultrasensitive method to monitor the oxidation metabolism of tumor tissues, enabling the early diagnosis of inflammation, ultrasensitive detection and further imaging‐guided surgery of cancers.^[^ ^8^ ^]^ Simultaneously, the energy transfer between the long‐duration CL reaction and PSs also provides the approach to the generation of cytotoxic species for the realization of tumor cell apoptosis and inhibition of tumor growth, enabling the in vivo targeted therapy.^[^ ^4^, ^5^, ^8^ ^]^ Nevertheless, there are still several concerns about these in vivo self‐illumination in imaging and therapy: 1) The tissue scattering and absorption may limit the optical penetration depths of short‐wavelength CL; 2) There are diversified limitations of classical materials, such as low CL quantum yield (CL QY), tedious preparation, low solubility, and high cost, etc; 3) It is still in doubt how the endogenous weak CL emission can achieve so valid therapeutic effect.

**Figure 1:**
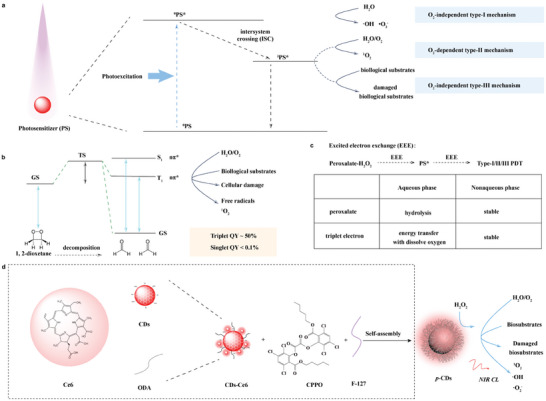
The mechanism of active type I/II/III photosensitizers and design of *p*‐CDs. a) Schematic illustration of different photochemical routes of photosensitizer (PSs). b) Schematic illustration of chemical route and approximate empirical energies for the 1, 2‐dioxetane and their decomposition of two formaldehyde molecules in the ground state (GS) or in the excited singlet (n, π*) (S_1_) and excited triplet (T_1_) states. TS: transition state structure. c) Schematic illustration of the possible excited electron exchange in the CL‐assisted photochemical reaction and corresponding environment effect. d) Schematic illustration of the preparation of the TME‐responsive *p*‐CDs for oncology imaging and synergistic dynamic therapy.

Conventionally, the classical photodynamic therapy involves the O_2_‐independent type‐I photochemical reaction that employ the e^−^/H^+^ transfer to generate the ·OH and ·O_2_^–^, and O_2_‐dependent type‐II photochemical reaction that is driven by electron spin exchange to generate ^1^O_2_ (**Figure** **1**a). ^[^ ^9^ ^]^ And the generation of excess cytotoxic species in cells can activate the apoptosis signaling pathway. However, the hypoxia of TME in a solid tumor may significantly affect the killing effect of photosensitive medicines upon tumor cells, and the activation between ROS and substrates in cells (lifetime of ≈1 ns and diffusion distance of ≈100 nm) may seriously emasculate the photochemical reaction, eliminating the cancer therapeutic effect. Recently, Sibata et al. have predicted O_2_‐independent type‐III photochemical reaction, in which the O_2_‐independent type‐III photochemical reaction can promote the energy transfer between the photosensitizer and biological substrates of proteins, nucleic acids, and other biomacromolecules, directly inducing the cell apoptosis.^[^ ^10^ ^]^ Yao et al. have approved the concept and examples of type‐III PSs for the enhanced anticancer efficiency.^[^ ^11^ ^]^ In these works, the electron exchange between the biomacromolecules and excited photosensitizer molecules has been demonstrated and the excited triplet electron can directly destroy the RNA molecules, enabling the apoptosis of tumor cells under hypoxia and even without oxygen. Ideally, in a 1,2‐dioxetanes‐related CL reaction (e.g., peroxalate‐H_2_O_2_), the uncatalyzed decomposition of 1,2‐dioxetane and its derivatives have been approved to generate the excited singlet and triplet carbonyl compounds (Figure 1b). Wherein, the low singlet quantum yield of <0.1% and high triplet quantum yield of ≈50% evoke the transformation with a very ideal model for photochemical reaction.^[^ ^12^ ^]^ With the photodynamic and CL mechanism, it is automatically proposed the combination of excited singlet/triplet electrons from a CL reaction and type‐II/III photochemical reaction to achieve efficient CL emission and dynamic therapy, ensuring a high‐resolution oncology imaging and enhanced anticancer efficiency.

Inspired by such findings, we have investigated the energy transfer mechanism between fluorophores and peroxalate‐H_2_O_2_ reaction and utilized the excess excited singlet/triplet electrons in CL to realize oncology imaging and synergistic dynamic therapy. As a proof‐of‐concept, the hydrophobic carbon nanodots (CDs)‐loaded chlorin e6 (CDs‐Ce6) are designed and integrated with peroxalate molecules (Figure 1c), and thus the as‐prepared polymer carbon nanodots (*p*‐CDs) exhibit novel tumor microenvironment‐responsive long‐persistent near‐infrared CL emission and photochemical progression (Figure 1d), evoking the in vivo biological imaging in tumor tissues and anticancer efficiency. Mechanistically, the excess ROS from the high level of oxygen metabolism in TME can trigger the chemically initiated electron exchange, and the hydrophobic CDs can promote triplet electron exchange between Ce6 and peroxalate‐derived 1, 2‐dioxetanes, evoking the generation of excess excited triplet electrons. As a result, the production of excess excited singlet/triplet electrons will successively lead to the long‐persistent near‐infrared (*NIR*) CL emission of Ce6, type I/II photochemical production of ·OH, ·O_2_^–^, and ^1^O_2_, and type III photochemical damage of maladjusted biomacromolecules in tumor cells (Figure 1d), ensuring the in vivo biological imaging and apoptosis of tumor cells. These nanoplatforms can perfectly circumvent the TME features and improve the therapeutic effect of classical PDT, providing a new sight into the design of multifunctional theragnostic nanoplatforms.

## 2 Result and Discussion

**Figure 2:**
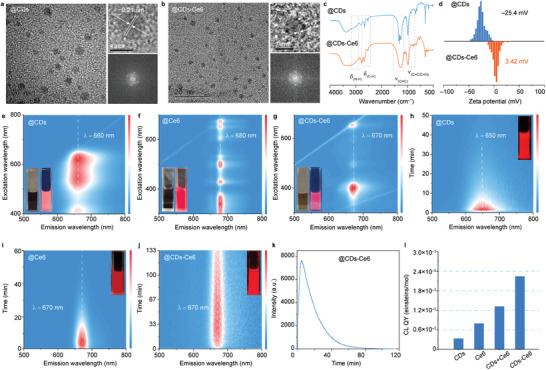
Structure and optical properties of the CDs and CDs‐Ce6. a,b) TEM, High‐resolution TEM (HRTEM) images and selected area electron diffraction (SAED) patterns of the CDs (a) and CDs‐Ce6 (b). c) FT‐IR spectra of the CDs and CDs‐Ce6. d) Zeta potentials of the CDs and CDs‐Ce6 solution. e–g) Excitation‐emission matrix of the CDs (e), Ce6 (f) and CDs‐Ce6 (g). h–j) Time‐resolved CL emission spectra of the CDs (h), Ce6 (i), and CDs‐Ce6 (j). k) CL intensity decay of the CDs‐Ce6 in CPPO+H_2_O_2_ solution. l) CL QYs of the CDs, Ce6, CDs+Ce6 and CDs‐Ce6.

In this work, the CDs with hydrophobic side chains are employed to load the PSs of Ce6 for the promotion of triplet electron exchange. The deep‐red fluorescent CDs are synthesized with citric acid and urea as precursors in N, N‐dimethylformamide (DMF) via solvothermal reaction,^[^ ^14^ ^]^ and the CDs‐loaded Ce6 (CDs‐Ce6) are further prepared with the CDs, Ce6 and amphiphilic octadecylamine.^[^ ^14^ ^]^ Transmission electron microscopy (TEM) images depict the well‐dispersed quasi‐spherical morphologies of CDs and CDs‐Ce6, featuring with the size distribution ≈2.19 and 2.45 nm, respectively (**Figure** **2**a,b; Figure S1, Supporting Information). The high‐resolution TEM (HRTEM) images present a consistent lattice fringe with a spacing of 0.21 nm, and the corresponding selected area electron diffraction (SAED) patterns indicate the (002) and (101) faces of graphitic carbon. Surface characterization, encompassing Fourier transform infrared spectroscopy (FT‐IR), nuclear magnetic resonance spectroscopy (NMR), X‐ray photoelectron spectroscopy (XPS), X‐ray diffraction (XRD), and zeta potential investigations, elucidate the chemical composition. The identical XRD patterns of CDs and CDs‐Ce6 emphasize their analogous graphitic carbon core (Figure S2, Supporting Information). In FT‐IR spectra (Figure 2c), both CDs and CDs‐Ce6 exhibit the peaks associated with –NH_2_, –CH_2_–, and –CH_3_ vibrations, denoting their similar functional groups. Notably, the CDs‐Ce6 display additional features, including a shoulder peak at ≈1570 cm^−1^ (N–H bending vibration of the amide bond) and a decreased peak at ≈3200 cm^−1^ (N–H stretching vibration of amino groups),^[^ ^15^ ^]^ confirming the successful Ce6 conjugation. Validation through ^1^H and ^13^C NMR spectra further affirm the conjugation. The ^1^H NMR spectrum of CDs‐Ce6 reveals a signal at 7–8 ppm corresponding to O═C–N– (Figure S3a, Supporting Information), which is consistent with the FT‐IR results. The ^13^C NMR spectrum of CDs‐Ce6 provides the clear evidence of aromatic H signal (Figure S3b, Supporting Information), serving as an additional confirmation of the successful conjugation between CDs and Ce6. The XPS spectra of both CDs and CDs‐Ce6 illustrate the presence of carbon, nitrogen, and oxygen elements. The high‐resolution C1s spectra reveal the similar peaks associated with C–C/C═C, C–C/C–N, and C═O groups, while the high‐resolution O1s spectra display comparable signals for carbonyl O and quinone O. These findings collectively indicate the sp^2^/sp^3^ hybrid framework and functional groups in the CDs and CDs‐Ce6.^[^ ^16^ ^]^ Similarly, the high‐resolution N1s spectra unveils the presence of pyrrole N and graphitic N in the CDs and CDs‐Ce6, with an additional peak attributed to amino N in CDs‐Ce6 (Figure S4, Supporting Information).^[^ ^17^ ^]^ These results confirm the successful conjugation of CDs‐Ce6, substantiating the coupling of the CDs with Ce6. Additionally, the zeta potential of CDs‐Ce6 is measured as 3.42 mV, a notable deviation from the initial CDs with a zeta potential of −25.4 mV (Figure 2d). Interestingly, the zeta potential of CDs‐Ce6 aligns closely with that of Ce6, which is recorded at 1.63 mV (Figure S5, Supporting Information). This observation supports the notion of electrostatic interaction‐related conjugation, emphasizing the role of electrostatic forces in the successful coupling of CDs and Ce6.

Subsequently, the optical properties of CDs, Ce6 and CDs‐Ce6 are compared. For the initial CDs and Ce6, the bright deep‐red/*NIR* PL emission with a maximal emission wavelength at 660 nm and 680 nm can be observed (Figure 2e,f). With the conjugation of Ce6, the CDs‐Ce6 present obviously different excitation‐independent emission (Figure 2g) and the maximal emission is consistent with the initial Ce6. Therefore, the photoluminescence quantum yield (PL QY) of CDs and CD‐Ce6 can be calculated as 37.37% and 17.34%, respectively, by the reference method (Figure S6a,b, Supporting Information), which verifies the existence of energy transfer between CDs and Ce6. Similarly, the time‐resolved fluorescence intensity reveals the PL lifetime of 3.84 ns for the CDs and 5.92 ns for the CDs‐Ce6 (Figure S7, Supporting Information), providing another evidence of direct electron transfer between the CDs and Ce6. Meanwhile, the Ce6 exhibit three absorption bands ranging from UV to deep‐red region (Figure S8, Supporting Information), and there is an overlap between the PL emission of CDs and absorption of Ce6, enabling the possible Förster resonance energy transfer. Compared with the CDs solution (Figure 2e), the Ce6 and CDs‐Ce6 present narrow PL emission band. Notably, when added into the solution containing bis(2,4,5‐trichloro‐6‐carbopertoxyphenyl) oxalate (CPPO) and H_2_O_2_, all the CDs, Ce6 and CDs‐Ce6 can emit bright and persistent *NIR* CL emission (Figure 2h–j). However, the initial CL intensity and the CL lifetime of the CDs‐Ce6 are far longer than the Ce6. Thus, the time‐resolved CL intensity of the CDs, Ce6, CDs+Ce6 and CDs‐Ce6 are further compared. As shown in Figures S9–S12 (Supporting Information), all these fluorophores can present long‐persistent deep‐red CL emission in the peroxalate‐H_2_O_2_ reaction while only the CL lifetime of CDs‐Ce6 can exceed 120 min (Figure 2k), which is far longer than the lifetime of 20, 60, and 83 min for the CDs, Ce6, and CDs+Ce6. Since the fluorophore concentrations mainly determine the initial CL intensity, there is little influence on the CL spectra and intensity decay (Figure S13, Supporting Information). With the CL reaction of lucigenin‐H_2_O_2_ as a reference (Figure S14, Supporting Information), the corresponding CL QYs of CDs, Ce6, CDs+Ce6 and CDs‐Ce6 can be calculated as high as 3.336 × 10^−4^, 7.983 × 10^−4^, 1.319 × 10^−3^ and 2.239 × 10^−3^ einsteins mol^−1^ (Figure 2l), underscoring the promotion for CL emission from the conjugation of Ce6.^[^ ^12^, ^13^, ^17^ ^]^

**Figure 3:**
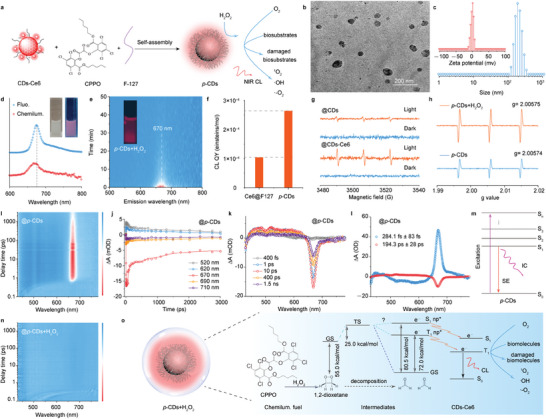
Synthesis and physicochemical properties of the *p*‐CDs. a) Design and synthesis of the *p*‐CDs and the triplet electron exchange for long‐persistent CL emission and type‐I/II/III photochemical reaction. b) TEM image of the *p*‐CDs. c) Dynamic light scattering (DLS) and zeta potentials of the *p*‐CDs. d) Normalized fluorescence and CL spectra of the *p*‐CDs aqueous solution. e) Time‐resolved CL emission spectra of the *p*‐CDs aqueous solution after adding H_2_O_2_. f) CL QYs of the Ce6@F127 and *p*‐CDs. g) ESR spectra of the CDs and CDs‐Ce6 under illumination or dark for the signal of ^1^O_2_. h) ESR spectra of the *p*‐CDs under illumination and *p*‐CDs+H_2_O_2_ under dark. i‐m) 3D transient absorption (TA) spectra for the *p*‐CDs expressed in ΔOD (the change of absorption intensity after excitation) as a function of delay time and probe wavelength (i), TA spectra of *p*‐CDs at indicated delay times (j), kinetic traces at different probe wavelength of the *p*‐CDs (k), Global fitting TA results of *p*‐CDs (l), and Schematic illustration of the luminescence of *p*‐CDs (m). n) 3D TA spectra for the *p*‐CDs+H_2_O_2_ expressed in ΔOD as a function of delay time and probe wavelength. o) Schematic illustration of the electron exchange mechanism in the CL emission from *p*‐CDs–peroxalate–H_2_O_2_ system.

On account of the splendid long‐persistent CL emission, the CDs‐Ce6 and CPPO molecules are integrated with amphiphilic polymeric conjugate of F127 to prepare the ROS‐responsive chemiluminescent *p*‐CDs (**Figure** **3**a). The *p*‐CDs nanoprecipitates appear as particles with a hydrodynamic diameter of ≈100 nm (Figure 3b,c). The hydrophobic CDs‐Ce6 can promote the self‐assembly of F127 (Figure S15, Supporting Information) and the introduction of surfactant F127 endows the *p*‐CDs with a near‐zero zeta potential of −0.57 mV and hydrophilic groups in FT‐IR spectrum (Figure S16, Supporting Information), evoking the excellent dispersion of *p*‐CDs in aqueous solution. Upon transformation of CDs‐Ce6 into *p‐*CDs in aqueous medium, the PL and CL emission maintains a similar peak at 670 nm (Figure 3d), and absorption peaks ≈405 and 650 nm remain consistent (Figure S17, Supporting Information), implying the successful self‐assembly of CDs‐Ce6 and peroxalate molecules. It should be emphasized that the amount of peroxalate, H_2_O_2_ and F127 have little influence on the NIR CL emission (Figures S18 and S19, Supporting Information). Notably, the CL emission peak exhibits minimal alteration compared with the CDs‐Ce6 (Figure 3e), approving the CL emitters of Ce6 molecules. These findings underscore the superior CL performances of the CDs‐Ce6. Since the aqueous environment has a bursting effect on the peroxalate‐H_2_O_2_, the CL duration of *p*‐CDs in aqueous solution remains almost 25 min, and the CL QY can be calculated as high as 2.264 × 10^−4^ einsteins mol^−1^ (Figure 3f), which is twofold of the Ce6@F127 (Figure S20, Supporting Information). Considering the feature of easy hydrolysis in peroxalate molecules (Figure S21, Supporting Information), the *p*‐CDs are stored in an anaerobic state under light shielding and refrigeration, endowing the stable optical properties of *p*‐CDs for ≈7 days. Meanwhile, the CDs‐Ce6 and *p*‐CDs exhibit the excellent capability to produce the cytotoxic species of ^1^O_2_ as the initial Ce6, which can be demonstrated by the electron paramagnetic resonance (ESR) (Figure 3g) and singlet oxygen sensor green (SOSG) after light illumination (Figure S22, Supporting Information). The excess generation of ^1^O_2_ implies the excited triplet electron orbits in CDs‐Ce6. Similarly, the CDs‐Ce6 and *p*‐CDs also can produce ·OH and ·O_2_^−^ under illumination as similar as the initial CDs (Figure S23, Supporting Information). Thus, the as‐prepared *p*‐CDs can successively achieve the production of ·OH, ·O_2_^−^ and ^1^O_2_ under illumination (Figure 3h), enabling the possible type‐I/II PDT. To further investigate the energy transfer mechanism, the transient absorption (TA) of the *p*‐CDs and *p*‐CDs+H_2_O_2_ are compared. As illustrated in Figure 3i to 3m, the strong negative features from 645 to 700 nm correspond to ground state bleaching (GSB) and stimulated emission (SE) with peak centered at 670 nm,^[^ ^16^ ^]^ which is consistent with the steady‐state fluorescence and absorption spectra of Ce6. The weak positive features from 440 to 640 nm correspond to the excited state absorption (ESA). The ΔOD mainly presents the delay peak at 1–1000 ps, indicating the intrinsic excitation of exciton, and the global fitting results demonstrate two principal decay associated difference spectra of *p*‐CDs. The fitted lifetimes of 284.1 fs and 194.3 ps are ascribed to two relaxation channels of internal conversion (IC) and SE pathways.^[^ ^8^, ^16^ ^]^ Interestingly, after adding H_2_O_2_ into the *p*‐CDs aqueous solution, the SE channel of the *p*‐CDs disappear (Figure 3n), implying the production of excess excited electron in the CL process.

In previous reports,^[^ ^12^, ^13^, ^17^ ^]^ the CL produced by the reaction between fluorophores and peroxalate–H_2_O_2_ is known as chemically initiated electron exchange luminescence (CIEEL).In the CL process, the oxidation reaction between the peroxalate and H_2_O_2_ can spontaneously generate the energetic intermediates (such as 1, 2‐dioxetanedione), and the excited electron exchange between the intermediates and fluorophores will lead to the excitation of fluorophores.^[^ ^4^, ^17^ ^]^ However, because the (n, π*) triplet and singlet excited states of formaldehyde are 72.0 and 80.5 kcal mol^−1^ less stable than the ground state (the reaction energy of 1,2‐dioxetane dissociation is 55.0 kcal mol^−1^, and the activation energy for fragmentation is 25.0 kcal mol^−1^), the triplet excited state path is open for dissociation,^[^ ^13^, ^18^ ^]^ resulting in the efficient formation of triplet excited carbonyl compounds and much lower yields of singlet excited products. Considering the hydrophobic surface of the CDs‐Ce6, the successful conjugation can improve the singlet/triplet electron exchange between Ce6 and the excited triplet carbonyl compounds, evoking excess excited electron for long‐persistent *NIR* CL emission (Figure 3o).

**Figure 4:**
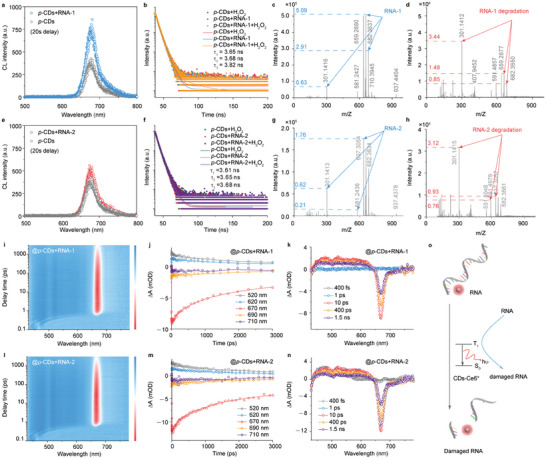
Triplet electron exchange between the *p*‐CDs and RNA molecules. a) CL spectra of the *p*‐CDs and *p*‐CDs+RNA‐1. b) Time‐resolved PL intensity decay of the *p*‐CDs, *p*‐CDs+RNA‐1 and RNA‐1+*p*‐CDs+H_2_O_2_ strand. c‐d) HRMS [M]^−^ of the initial RNA‐1 strand (c) and RNA‐1+H_2_O_2_+*p*‐CDs strand (d). e) CL spectra of the *p*‐CDs and *p*‐CDs+RNA‐2. f) Time‐resolved PL intensity decay of the *p*‐CDs, *p*‐CDs+RNA‐2 and *p*‐CDs+RNA‐2+H_2_O_2_. g,h) HRMS [M]^−^ of the initial RNA‐1 strand (g) and the RNA‐2+*p*‐CDs+H_2_O_2_ strand (h). i‐k) TA expressed in ΔOD as a function of wavelength and delay time (i), TA kinetic traces probed at different wavelengths (j), and TA spectra at indicated delay time from 1 to 1000 ns (k) for the *p*‐CDs. l–n) TA expressed in ΔOD (l), TA kinetic traces probed at different wavelengths (m), and TA spectra at indicated delay time from 1 to 1000 ns (n) for the *p*‐CDs+RNA‐2. o) Schematic illustration of the RNA damage from the triple electron of *p*‐CDs.

Since the conjugation of CDs and Ce6 can promote the electron exchange between Ce6 and intermediates, it is potential to ascertain the excess excited electron of *p*‐CDs+H_2_O_2_ to achieve the type‐I/II photochemical reaction and type‐III photochemical damage of biological substrates, realizing the synergistic dynamic therapy. To validate the hypothesis, two short strands of RNA biomacromolecules (RNA‐1 and RNA‐2) are treated with the *p*‐CDs+H_2_O_2_ in aqueous solution and further analyzed. With the comparation between the CL emission from the *p*‐CDs+H_2_O_2_ and *p*‐CDs+RNA‐1+H_2_O_2_, it can be found that there is a minimal change in CL spectra with an obvious decrease in CL intensity after adding the *p*‐CDs+H_2_O_2_ into RNA‐1 molecules (**Figure** **4**a), approving the energy transfer between the *p*‐CDs+H_2_O_2_ reaction and RNA‐1 molecules. Meanwhile, the *p*‐CDs+RNA‐1, and *p*‐CDs+RNA‐1+H_2_O_2_ present slight longer lifetime than pure *p*‐CDs+H_2_O_2_ (Figure 4b), and the UV–vis absorption of *p*‐CDs slightly decrease after adding RNA‐1 (Figure S24, Supporting Information), implying that the RNA‐1 molecules can eliminate the H_2_O_2_‐related nonradiative transition under photoexcitation. On the condition, the RNA and *p*‐CDs+RNA‐1+H_2_O_2_ are directly measured with the high‐resolution mass spectrometry (HRMS). The corresponding HRMS [M]^−^ strength of the strand RNA‐1 decrease from 5.09 × 10^5^ to 0.85 × 10^4^ after adding the *p*‐CDs+H_2_O_2_ (Figure 4c, d), implying that ≈97% RNA‐1 molecules have degraded. Similarly, the CL intensity of *p*‐CDs+H_2_O_2_ decrease after adding the RNA‐2 molecules (Figure 4e), the *p*‐CDs+RNA‐2, and *p*‐CDs+RNA‐2+H_2_O_2_ present slight longer lifetime than the pure *p*‐CDs+H_2_O_2_ (Figure 4f), and the HRMS [M]^−^ strength of RNA‐2 decreases from 1.76 × 10^5^ to 0.93 × 10^4^ and approves that ≈95% RNA‐2 molecules have degraded after the RNA‐2 molecules are treated with the *p*‐CDs+H_2_O_2_ (Figure 4g,h). These surveys clearly demonstrate that the production of *p*‐CDs+H_2_O_2_ reaction can directly destroy the RNA biomacromolecules.

To further understand the inner energy transfer mechanism between the RNA molecules and *p*‐CDs+H_2_O_2_ reaction, the femtosecond time‐resolved transient absorption (TA) experiments are conducted. As shown in Figure 4i–n, the *p*‐CDs and *p*‐CDs+RNA‐1 and *p*‐CDs+RNA‐2 present almost the same TA performances, while the TA signal intensities of *p*‐CDs+RNA‐1 and *p*‐CDs+RNA‐2 are much lower than that of the *p*‐CDs, indicating that the number of excited electrons of the *p*‐CDs has changed under light illumination. The decreased TA intensity directly confirm the excited triplet electron transfer between the *p*‐CDs and RNA macromolecules as previous report (Figure S25, Supporting Information).^[^ ^10^ ^]^ Additionally, when the RNA‐1 or RNA‐2 molecules are added into the *p*‐CDs aqueous solution, the fluorescent sensing from SOSG probes reveals the less generation of ^1^O_2_ (Figure S26, Supporting Information) and indicates the competitive relationship between dissolved oxygen and RNA macromolecules, implying the excited triplet exchange between the RNA molecules and *p*‐CDs+H_2_O_2_ reaction. Thus, it is prospective for the hypothesis that the triplet electron exchange can directly occur between the RNA molecules and *p*‐CDs+H_2_O_2_ reaction and the energy transfer between the *p*‐CDs+H_2_O_2_ reaction and RNA molecules will directly lead to the degradation of RNA‐like biological substrates (Figure 4o).

**Figure 5:**
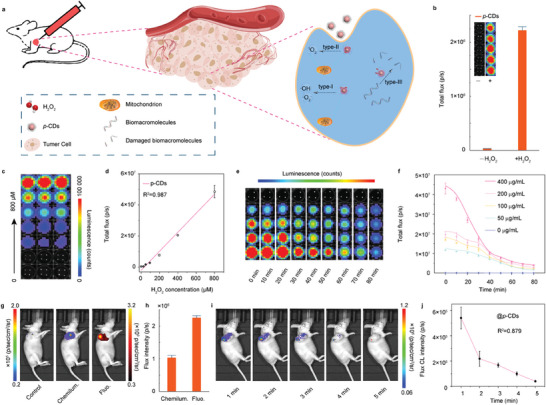
In vitro and in vivo biological imaging of the *p*‐CDs. a) Schematic representation of in vivo CL biological imaging and type‐I/II/III photodynamic therapy from *p*‐CDs. b) CL image and quantitative intensity of the *p*‐CDs with and without the presence of 50 µM H_2_O_2_ PBS solution. c,d) CL image (c) and corresponding quantitative intensities (d) of the *p*‐CDs captured in 0–800 µM H_2_O_2_ PBS solution. e,f) Time‐resolved CL images of the *p*‐CDs (0‐400 µg mL^−1^) captured in 50 µm H_2_O_2_ PBS solution (e) and quantitative intensity (f). g,h) CL and fluorescence images of the *p*‐CDs after intratumor injection (g) and quantitative intensities (h). i,j) Time‐resolved CL images (i) and quantitative CL intensity (j) of the *p*‐CDs after intratumor injection.

With the novel long‐persistent *NIR* CL emission and type‐I/II/III PDT, the *p*‐CDs are promising to be utilized as intelligent biomedicines to achieve TME‐responsive long‐duration biological imaging and synergistic dynamic therapy simultaneously (**Figure** **5**a). Typically, the high levels of metabolism and maladjusted biosynthesis intermediates in TME endow the tumor cell with the excess ROS and numerous biological macromolecules. Mechanistically, the excess ROS in tumor cells will trigger the *NIR* CL emission of *p*‐CDs to achieve the in vivo long‐persistent biological imaging, and the triplet electron exchange will successively lead to the type II photochemical production and type III photochemical damage of biomolecules, endowing the enhanced anticancer efficiency. On the condition, the in vitro and in vivo TME‐responsive CL emission of the *p*‐CDs are investigated with an in vivo imaging system (IVIS). Herein, the *p*‐CDs present far higher CL intensity in the presence of H_2_O_2_ (50 µm) (Figure 5b), elucidating the high sensitivity to the exogenous ROS. After adding the *p*‐CDs into H_2_O_2_ PBS solution, there is a well linear relationship between the CL intensity and H_2_O_2_ concentration with a limit of detection (LOD) of 0.18 µM (Figure 5c,d), indicating the capability of *p*‐CDs for sensitive ROS detection. When added into H_2_O_2_ PBS solution, the different concentration of *p*‐CDs present obviously different initial intensity with similar decay curve and the detectable CL signals can last for more than 80 min (Figure 5e,f), approving the potential of *p*‐CDs in long‐duration biological imaging.

Thus, the *p*‐CDs are further employed for the detection and diagnosis of the tumor tissues by the in vivo inflammatory imaging. As shown in Figure 5g,h, the tumor models are established and the in vivo bioimage of mouse models have been subsequently captured for 3 min after the injection of *p*‐CDs. As a result, both PL and CL signals can be captured after the tumor tissues are treated with the intratumor injection of *p*‐CDs. In comparison with the optical imaging established with the PL signals, the CL imaging present a maximal signal‐to‐noise ratio (SNR) of 25.98 dB for the excess ROS in tumor and reveal the distribution of endogenous H_2_O_2_ in the tumor tissue, providing an excellent approach to the analytes of the quantification and localization of the tumor tissues. With the continuous determination, the detectable in vivo CL signals present obvious decrease after the intratumor injection and the CL signals can last more than 5 minutes after the injection, implying the gradual exhaustion of endogenous H_2_O_2_ (Figure 5i,j). The long‐duration CL imaging endow the *p*‐CDs with the capability of diagnosis and further promising imaging‐guided surgical resection for cancer.

**Figure 6:**
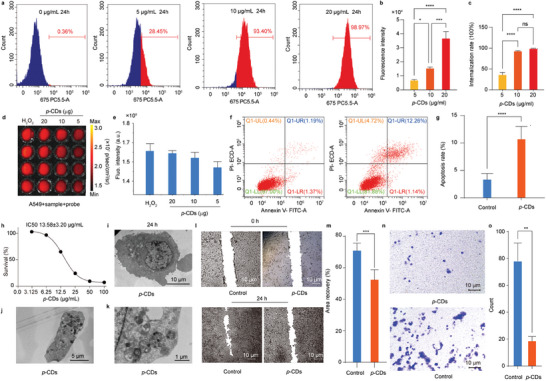
In vitro antitumor performance of the *p*‐CDs. a–c) The internalization of A549 cells treated with the *p*‐CDs (5, 10 and 20 µg mL^−1^) for 24 h through flow cytometry (a) and corresponding fluorescence intensity (b) and internalization rate (c). d,e) The production of ^1^O_2_ from fluorescence images (d) and corresponding intensities statistics (e) of the A549 cells treated with oxidative stress probes after adding H_2_O_2_ (20 µg mL^−1^) and *p*‐CDs (5, 10 and 20 µg mL^−1^). f,g) The cell apoptosis of A549 cells incubated without and with the *p*‐CDs (20 µg mL^−1^) in flow cytometry (f), and the corresponding apoptosis rate (g). h) Relative viabilities of A549 cells with the different concentrations of *p*‐CDs. i–k) TEM images of the A549 cells treated with the *p*‐CDs. l,m) The wound area (l) and corresponding recovery rate (m) of A549 cells after treated with the *p*‐CDs in wound healing assay. n,o) The photographs (n) and corresponding numbers (o) of migration A549 cells after treated with the *p*‐CDs in transwell assay.

With the *ROS*‐responsive type I/II/III photochemical reaction, the *p*‐CDs are promising to further serve as nanoplatform for cancer therapy. Herein, the in vitro antitumor efficiency of *p*‐CDs has been investigated. First, the internalization effect of A549 cells upon the *p*‐CDs is examined. After a same period of incubation, the A549 cells incubated with a higher concentration of *p*‐CDs illustrate a higher fluorescence intensity and thus present higher internalization rate (**Figure** **6**a). The corresponding statistical results clearly reveal that the *p*‐CDs can be ingested by the A549 cells through endocytosis, and the internalization efficiency is influenced by the concentration of *p*‐CDs (Figure 6b,c). Meanwhile, after incubated with the *p*‐CDs, the monitoring of oxidative stress probes in the A549 cells present higher fluorescence intensity, revealing that the *p*‐CDs can induce the oxidative stress effect of cells as similar as the oxidative stress reagent of H_2_O_2_ (Figure 6d,e). Thus, the survival influence of *p*‐CDs to cancer cells is further examined. As depicted in Figure 6f,g, the flow cytometry of the A549 cells incubated with the *p*‐CDs presents more apoptosis area and the statistical apoptosis rate clearly demonstrates the *p*‐CDs‐induced cell apoptosis, approving the in vitro antitumor activity of *p*‐CDs.

Whereas, the cell survival rates of tumor cells upon the *p*‐CDs are detected by the flow cytometry. As depicted in Figure 6h, the A549 cells present a IC50 of 13.58 ± 3.20 µg mL^−1^ when incubated with the *p*‐CDs, and the results obviously reveal the proliferation inhibition of *p*‐CDs on different tumor cells (Figure S27, Supporting Information). The corresponding TEM images of A549 cells reveal that the *p*‐CDs‐treated A549 cells present obvious breakage in membrane and there are numerous autophagosome in the cells (Figure 6i–k), approving the cell apoptosis induced by cytotoxic species. On the condition, the influence of *p*‐CDs on the migration capability of tumor cells has been tested by the cell scratch experiment. The wound area and corresponding recovery rates of the A549 cells after incubated with the *p*‐CDs approve that the *p*‐CDs can significantly inhibit the migration of tumor cells (Figure 6l,m). Similarly, the influence of *p*‐CDs on the invasion ability of tumor cells is tested by the transwell assay. The photographs and corresponding numbers of migration A549 cells after treated with the *p*‐CDs demonstrate that the *p*‐CDs can significantly inhibit the invasion ability of A549 cells (Figure 6n,o). Summarizing all these results, it can be revealed that the A549 cells can naturally ingest the *p*‐CDs and the *p*‐CDs can produce the excess cytotoxic ^1^O_2_ in the tumor cells, endowing the apoptosis of tumor cells and thus enabling the in vitro inhibitory effect on tumor migration and invasion.

**Figure 7:**
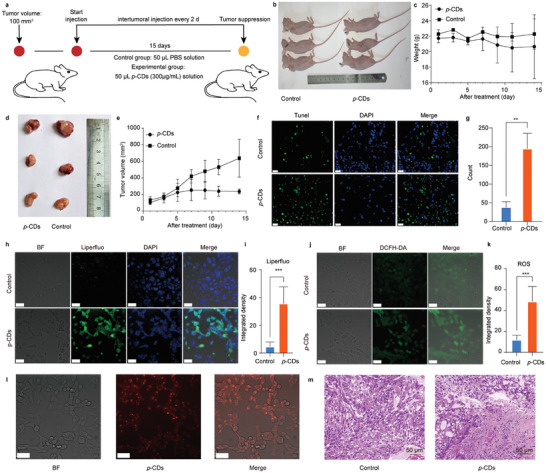
In vivo antitumor performance of the *p*‐CDs. a) Schematic illustration of the in vivo therapeutic application of *p*‐CDs. b) Representative digital photos of the mice in the control and *p*‐CDs‐treated groups. c) Body weights of the nude mice recorded every 2 days for the control and *p*‐CDs‐treated groups. d,e) The corresponding digital photo (d) and representative quantitative volumes (e) of the tumor tissues. f,g) The TUNEL and DAPI labeling assay (f) and quantitative analysis (g) of cell apoptosis in paraffin sections. h,i) The confocal microscopy images (h) and lipid peroxidation levels (i) in the A549 cells incubated with Liperfluo. Scale bars, 100 µm. j,k) The corresponding confocal microscopy images (j) and ROS levels (k) in the A549 cells incubated by DHE probes. Scale bars, 100 µm. l) Confocal microscopy of the A549 cells incubated with the *p*‐CDs. Scale bars, 100 µm. m) The corresponding microscopic images of H&E‐stained sections of the tumor tissues from the killed mice.

As shown in **Figure** **7**a, the in vivo anticancer capability of *p*‐CDs has been evaluated in the tumor‐bearing mice. In detail, the mice are randomly divided into control group and *p*‐CDs‐treated group when the tumor volume reached 100 mm^3^. The mice in the *p*‐CDs group are treated with 50 µL *p*‐CDs PBS solution (300 µg mL^−1^) by intertumoral injection every 2 days and the mice are euthanized at the volume of total tumor achieved to ≈1000 mm^3^. Finally, the tumor tissues are stripped via surgical resection for further analysis. As a result, the body weights of the mice in control and *p*‐CDs‐treated groups present almost the same change within time in which the fluctuate may be induced by the tumor‐caused uneven nutritional intake (Figure 7b,c). However, in comparison with the rapid growth of tumor tissues in the control group, the tumor volumes in the *p*‐CDs‐treated mice increase extremely slowly (Figure 7d,e), thereby obviously indicating the in vivo antitumor capability of the *p*‐CDs.

On the condition, the in vivo antitumor effect of the *p*‐CDs is further investigated with the invaded A549 cells. The TUNEL assay shows that the numbers of TUNEL positive cells in the area of intertumoral injection significantly increase (Figure 7f). The result indicates that the relative expression intensity of TUNEL is significantly up‐regulated in the *p*‐CDs treated transplanted tumor tissues, approving that the *p*‐CDs can promote the cell apoptosis in transplanted tumors (Figure 7g). Meanwhile, the Liperfluo probes are employed to detect the lipid peroxidation levels in the A549 cells incubated with *p*‐CDs. The level of lipid peroxidation in the A549 cells that are treated with the *p*‐CDs is also significantly increased (Figure 7h,i). Similarly, the DCFH‐DA probes are used to detect the ROS levels in the A549 cells treated with the *p*‐CDs, and the result indicates that the ROS levels in the A549 cells are significantly up‐regulated after the incubation of *p*‐CDs (Figure 7j,k). In addition, the confocal laser fluorescence microscopy is used to test the in vivo internalization of *p*‐CDs in the A549 cells. As depicted in Figure 7l, the fluorescence microscopy images of the A549 cells present bright fluorescence signals after the injection of *p*‐CDs, clearly indicating that the A549 cells of tumor tissues have internalized the *p*‐CDs. Subsequently, the safety profile of *p*‐CDs administered to the mice is further investigated with the H&E‐stained sections of tumors ablated at Day 14. In comparison with the control group, the tumor of the *p*‐CDs treated mouse present more severe cell apoptosis or necrosis and the blood vessels in the tumor tissue are destroyed to the point of being almost invisible (Figure 7m). These surveys strongly validate that the mitochondrial pathway of apoptosis enable the *p*‐CDs to induce the cell apoptosis, endowing the *p*‐CDs with the novel in vivo antitumor capability.

Since the proposed CDs‐Ce6‐based system is somewhat inferior to the lanthanide‐doped nanoparticles in terms of emission wavelength and lifetime (Table S1, Supporting Information), the multifunctional theragnostic nanoplatform still exhibit novel potential in long‐lasting NIR bioimaging and biotherapy. However, there are still several necessary processing techniques for the clinical translation: 1) scalability to ensure consistent quality and performance, and cost‐effectiveness manufacturing processes to produce large quantities; 2) surface engineering and functionalization to achieve precise targeting, reduce aggregation, improve stability, and extend circulation time; 3) regulatory compliance including preclinical testing, toxicology studies, and clinical trials to ensure the safety of nanoplatform, and good manufacturing practices (GMP) to ensure product safety and quality; 4) stability and shelf life to ensure the activity and stability of nanoplatform over extended periods of time, and preserve the nanoparticles' structure and activity, facilitating long‐term storage and transportation. Therefore, the prospect of the CDs‐Ce6 system for the clinical application is still an enormous challenge.

## 3 Conclusion

In summary, the electron exchange mechanism has been demonstrated between the fluorophores and peroxalate–H_2_O_2_ reaction, endowing the improvement of the CL lifetime, the promotion for the photochemical production of ^1^O_2_ under hypoxia and the photochemical damage of biomacromolecules. As a result, a theragnostic nanoplatform with CL imaging and synergistic cancer therapy has been developed. The as‐prepared *p*‐CDs present novel TME stimuli‐responsive long‐duration CL imaging, enabling high detection sensitivity and specificity, superior imaging quality, and satisfactory safety for in vitro and in vivo tumor diagnostics. Meanwhile, the TME‐related ROS and maladjusted biosynthesis intermediates as active substances can effectively trigger the type I/II/III photochemical reaction of *p*‐CDs and thus activate the apoptotic pathway of tumor cells, endowing the *p*‐CDs with the excellent capability of cancer therapy. With the multiple advantages of simultaneous diagnosis and treatment, the *p*‐CDs are promising to be developed as the new nanomedicines for future clinical translation.

## 4 Experimental Section

### Materials and Animal

Citric acid (C_6_H_8_O_7_, 99.9%), Urea (CON_2_H_4_, 99.9%), Chlorine e6 (Ce6, C_34_H_36_N_4_O_6_, 94%), Pluronic F‐127 (Poly(ethylene glycol)‐block‐poly(propylene glycol)‐block‐poly(ethylene glycol) diacrylate, F127), Octadecylamine (C_18_H_39_N, 97%), N, N‐Dimethylformamide (C_3_H_7_NO,99.0%, DMF) and Bis(2,4,5‐trichloro‐6‐carbopertoxyphenyl) oxalate (CPPO, 98%) were purchased by Aladdin Reagent Co., Ltd (Shanghai, P. R. China). Ethyl alcohol (EtOH, 99.9%) and hydrogen peroxide (H_2_O_2_, 30 wt.%, 99%) was purchased from Sinopharm Chemical Reagent Co., Ltd (Shanghai, P. R. China).

### Animal and Ethical Approval

The specific‐pathogen‐free grade BALB/c Nude mice were purchased from Bei Jing Vital River. All animal experiments were approved by The First Affiliated Hospital of Zhengzhou University under Protocol No. 2019‐KY‐008.

### Synthesis of the CDs‐Ce6

Typically, 1 g citric acid and 2 g urea were dispersed into 10 mL N, N‐dimethylformamide (C_3_H_7_NO, DMF). The mixture was added to a Teflon‐lined stainless steel autoclave (20 mL). The sealed autoclave vessel was then placed in an electric furnace set at 180 °C and maintained for 10 h. After adding same volume of ethanol into the resulting solution, the precipitate was obtained by centrifugation (8000 r min^−1^, 10 min), and further purified by silica gel column chromatography with DMF as eluent. The as‐prepared CDs solution was spin evaporated to obtain black CDs powder at 80 °C. Then, 0.01 g CDs, 0.05 g octadecylamine and 0.01 g Ce6 were mixed in 5 mL DMF solution with stirring under 80 °C for 12 h. The obtained solvent was extracted with dichloromethane to obtain CDs‐Ce6 solution. The final CDs‐Ce6 product was collected from the dichloromethane solvent by rotary evaporator for characterization and further using.

### Preparation of the p‐CDs

To prepare the chemiluminescent *p*‐CDs, the CDs‐Ce6 (1 mg mL^−1^), Pluronic F‐127 (30 mg mL^−1^) and bis[2,4,5‐trichloro‐6‐aminophenyl] oxalate (CPPO, 3 mg mL^−1^) were dissolved into dichloromethane by ultrasonic vibration and stirring. Then, 1 mg CDs‐Ce6 (1 mg mL^−1^), 30 mg F‐127 (30 mg mL^−1^), and 1 mg CPPO (1 mg mL^−1^) were homogeneously mixed in a flask. After removing the solvent with a vacuum pump, the dried products were dissolved in Milli‐Q water or PBS to prepare the *p*‐CDs solution at a concentration of 300 µg mL^−1^.

### In Vitro CL Characterization of the p‐CDs

For the quantitative analysis, 50 µL *p*‐CDs (300 µg mL^−1^) were placed in a black 96‐well ELISA plate, then 50 µL H_2_O_2_ (0, 100, 200, 400, and 800 µmol L^−1^) (Sigma‐Aldrich, 323 381) was added. After quick shaking, the plate was immediately transferred into the in vivo imaging system (Lumina Series III, Xenogen, USA) to acquire luminescent signals (exposure time: 30 s, open filter) at every 5 min within 80 min.

### In Vivo CL Characterization of the p‐CDs

For the CL characterization analysis in vivo, 100 µL *p*‐CDs (300 µg mL^−1^) were injected into the tumor of the mice after the tumor‐bearing mice were successfully established. Then, the mice were immediately transferred into the in vivo imaging system (Lumina Series III, Xenogen, USA) to acquire luminescent signals and fluorescence signal. At the same time, the persistence of the fluorescence signal was observed within 5 minutes.

### Internalization of the p‐CDs In Vitro

The A549 cells were seeded in 12‐well plates (1 × 10^5^ cells well^−1^) and incubated for 24 h. Then, 5, 10, and 20 µg mL^−1^ *p*‐CDs were added and incubated for 24 h. The cells were harvested and washed twice times with 4 °C phosphate‐buffered saline. Then, cells were resuspended in 1 mL PBS gently vortex, and analyzed by flow cytometry (CytoFLEX, Beckman Coulter, USA) at excitation wavelength of 488 nm and emission wavelength of 675 nm. For fluorescence internalization analysis, the A549 cells were seeded in Confocal Dishes (2 × 10^5^ cells well^−1^) for 24 h, then 20 µg mL^−1^ *p*‐CDs were added and incubated for 24 h. The cells were washed twice times with 4 °C PBS, and were analyzed by Spinning Disc Confocal Microscope (Ultraview VOX, Perkin Elmer, USA) at excitation wavelength of 488 nm and emission wavelength of 675 nm.

### ROS Measurement in Cells

A549 cells were seeded in Confocal Dishes (2 × 10^5^ cells well^−1^) for 24 h, and then exposed to *p*‐CDs (10 µg mL^−1^ based on CDs‐Ce6) for 24h. 2,7‐Dichlorodi‐hydrofluorescein diacetate (D6883, Sigma–Aldrich, USA) was added at final concentration of 10 µm and incubated for 30 min at 37 °C in the dark. Cells were washed twice with 4 °C PBS and analyzed by Spinning Disc Confocal Microscope (Ultraview VOX, Perkin Elmer, USA) at 488 nm.

### Lipid Peroxidation Analysis

The A549 cells were seeded in Confocal Dishes (2 × 10^5^ cells well^−1^) for 24 h, then 20 µg mL^−1^ *p*‐CDs were added and incubated for 24 h. Remove the medium and washed the cells one time with serum‐free medium. Add 200 µL Liperfluo (L248, Dojindo, Japan) working fluids at 1 µm and incubate in a 5% CO_2_ incubator at 37 °C for 30 min. Remove the solution and wash two times with 200 µL HBSS and then analyzed by Spinning Disc Confocal Microscope (Ultraview VOX, Perkin Elmer, USA) at excitation wavelength of 488 nm and emission wavelength of 525 nm.

### Apoptosis Detection

Apoptosis was performed by FITC Annexin V Apoptosis Detection Kit (BD Bioscience, San Jose, CA, USA, 556 547). In brief, A549 (1 × 10^5^ cells well^−1^) cells were seeded in 12‐well plates for 24 h, then the cells were treated with the *p*‐CDs (20 µg mL^−1^) and incubated for 24 h. The cells were harvested and washed twice times with 4 °C PBS and centrifuged (250 × g, 5 min, 4 °C). Then, cells were resuspended in 1 × binding buffer to a final concentration of 1 × 10^6^ mL^−1^. The annexin V (5 µL) and PI (5 µL) were added, and then incubated for 15 min at room temperature in the dark. The apoptosis rate was analyzed by flow cytometry (CytoFLEX, Beckman Coulter, USA) at an excitation wavelength of 488 nm.

### In Vitro Cytotoxicity

The A549 cells were seeded in 96‐well plates (5000 cells well^−1^) for 24 h. Then, the cells were treated with the varying concentrations of *p*‐CDs (3.125, 6.25, 12.5, 25, 50, 100 µg mL^−1^) and incubated for 24 h. Add 10 µL well^−1^ CCK‐8 (Dojindo, CK04) and incubated for 2 h at 37 °C. The optical density (OD) was measured at 450 nm (Epoch, Biotek, Winooski, VT, USA). Survival rate is calculated as: (OD treatment–OD blank)/(OD control–OD blank) × 100%, and the half‐limiting dose (IC50) was estimated.

### Wound Healing Assay

A549 cells were seeded in 12‐well plates (1 × 10^5^ well^−1^) and continue cultured to achieve 95% confluence, and then the wells were scratched with a 10 µL sterile pipette tip. The cells were followed treatment with *p*‐CDs (20 µg mL^−1^ based on CDs‐Ce6) for 24 h. Wound width was observed with a microscope (Leica, Wetzlar, Germany) after treatment at 0 h and 24 h. Scratch areas were calculated by ImageJ (National Institutes of Health, USA). The area recovery rate (%) is calculated by comparing the healing area before and after treatment.

### Invasion Assay

The upper chamber of Transwell (3422, Corning, USA) was pre‐coated with with 25 µL Matrigel matrix (354 248, Corning, USA). Add 200 µL cell suspension (1 × 10^5^ well^−1^) in FBS free RPMI 1640 medium with the *p*‐CDs (20 µg mL^−1^) to the upper chamber and the lower chamber was added with 600 µL complete medium (10% FBS). After incubation for 24 h, the invasive cells in the lower chambers were fixed by 4% paraformaldehyde and stained by 0.5% crystal violet solution. The stained cells were counted in 5 random fields with microscope (Leica, Germany) at 100 × magnification.

### Ultramicroanalysis of Cell Damage

A549 cells were seeded in 75 cm^2^ (1 × 10^6^ well^−1^) and cultured after the growth density reached ≈50%. Then, the *p*‐CDs (5 µg mL^−1^) were added and incubated for 24 h. After washed two times with PBS, the cell suspension was collected in 15 mL by cell scraping. Cells were collected by centrifugation at 1000 rpm for 10 min and fixed by glutaraldehyde for 24h. After centrifugation, the cells were pre‐embedded with agar and fixed after dehydration at room temperature, and after osmoval, they were embedded with 812 compound (90529‐77‐4, SPI, USA), and then polymerized in a 60 °C oven for 48 h. The resin block is used in the ultra‐thin microtome at 60–80 nm ultra‐thin section. The copper mesh was stained with 2% uranium acetate saturated alcohol solution in the dark for 8 min, and washed three times with 70% alcohol and ultrapure water. Then, the copper mesh was stained with 2.6% lead citrate solution to avoid carbon dioxide, after washed with ultrapure water for three times, the copper mesh was dried and observed under a transmission electron microscope after cleaning (HT7800, hitachi, Japan).

### Xenograft Tumor Growth and Therapy

After the six mice acclimatized for 2 weeks, the A549 cells (2 × 10^6^) in 200 µL RPMI 1640 medium (50% Matrigel) were injected into the under the right forelimb armpit of mice. Mice weight and tumor volume was monitored every 2 d, and the tumor volume was calculated as follows: ([short diameter]^2^ × long diameter)/2. The mice were randomly divided into control group and *p*‐CDs group when the tumor volume reached 100 mm^3^. The mice in the *p*‐CDs group were treated with the *p*‐CDs 50 µL (300 µg mL^−1^) by intertumoral injection every 2 d. The mice were euthanized at the volume of total tumor achieved to 1000 mm^3^.

### Tunel Staining

The fluorescent staining for tissue apoptosis was detected by In Situ Cell Death Detection Kit (11 684 817 910, Roche, USA). In briefly, the tissue sections were deparaffinized and permeabilization, and washed three times with PBS. After incubated by Proteinase K, added tunel working solution 100 µL (TDT: dUTP = 2:29) and incubated at 37 °C for 2 h in wet box. Coverslip with anti‐fade mounting medium and examined through upright fluorescence microscope (NIKON ECLIPSE TI‐SR, Nikon, Japan) at excitation wavelength 488 nm and emission wavelength 520 nm. The tunel positive cells were counted by ZEN software (Carl Zeiss, Germany).

### Haematoxylin and Eosin (H&E) Staining

After fixed in 4% formaldehyde for 48 h, the tumor tissues were cut into 5 µm‐thick sections. Then, the sections were dewaxed with gradient concentration xylene and alcohol, and stained with haematoxylin and eosin dye according to the reagent kit. After the sections were mounted, a digital slide scanner and slide viewer software (Pannoramic 250 and Case Viewer 2.4, 3D Histech, Hungary) was used to observe the histopathology of tumor.

## Conflict of Interest

The authors declare no conflict of interest.

## Supporting information



Supporting Information

## Data Availability

The data that support the findings of this study are available on request from the corresponding author. The data are not publicly available due to privacy or ethical restrictions.
