# Supplementary material for: Triplet Electron Exchange in Carbon Nanodots‐assisted Long‐persistent near‐infrared Chemiluminescence for Oncology Synergistic Imaging and Therapy
Source: Adv Sci (Weinh). 2024 Dec 11;12(5):2411898. doi: 10.1002/advs.202411898 (PMC11791938; doi:10.1002/advs.202411898)
Supplement: Supplementary file 1 — Supporting Information [file ADVS-12-2411898-s001.docx]

**Supporting Information**

**Triplet electron exchange in carbon nanodots-assisted long-persistent near-infrared chemiluminescence for oncology synergistic imaging and therapy**

*Run-Wei Song, Tian-Ci Jiang, Xue-Yang Zhang, Cheng-Long Shen^*^, Qing Lou, and Chong-Xin Shan**

R. W. Song, C. L. Shen, Q. Lou, C. X. Shan
Henan Key Laboratory of Diamond Optoelectronic Materials and Devices, Key Laboratory of Material Physics, Ministry of Education, and School of Physics and Laboratory of Zhongyuan Light, Zhengzhou University, Zhengzhou 450052, China.

T. C. Jiang

Department of Respiratory and Critical Care Medicine, The First Affiliated Hospital of Zhengzhou University, Zhengzhou 450052, China.

X. Y. Zhang

College of Public Health, Zhengzhou University 450052, Zhengzhou, China.

R. W. Song, T. C. Jiang, X. Y. Zhang

These authors contributed equally.

E-mail: phyclshen@zzu.edu.cn (C. L. Shen), cxshan@zzu.edu.cn (C. X. Shan)

**Characterization**

Transmission electron microscopy (TEM, JEOL-2010). The crystalline property was evaluated in a Bruker-D8 Discover X-ray diffractometer with the Cu Kα line (λ = 1.54 Å) as the irradiation source. The X-ray photoelectron spectroscopy (XPS) was measured on a Kratos AXIS HIS 165 spectrometer with a monochromatized Al KR X-ray source (1486.7 eV). Fourier transform infrared (FT-IR) spectroscopy was performed using a Thermo Scientific Nicolet iZ 10 spectrometer in the KBr tablets. Photograph was obtained from a D610 camera (Nikon, Japan). The fluorescence and chemiluminescence spectrum of was measured by a F-7000 spectrofluorometer (Hitachi, Japan). The absorption spectrum was measured by an UV/vis spectrophotometer (Hitachi, UH-4150). The fluorescence decay curves were measured by Horiba FL-322 using a 370 nm Nano-LED monitoring the related emission. The PL QYs were measured by the spectrophotometer (FLS1000). The ^1^H and ^13^C chemical shift of CDs and CDs-Ce6 were measured by nuclear magnetic resonance (NMR) (Bruker 400/600M AVANCEIII) in D_2_O and CDCl_3_. The photochemical free radicals and single oxygen were measured through electron spin resonance (ESR) (Bruker A300). The dynamic light scattering (DLS) and zeta potential was measured on Zeta sizer Nano ZS90 (Malvern). Transmission electron microscopy (TEM, JEOL-2010). The crystalline property was evaluated in a Bruker-D8 Discover X-ray diffractometer with the Cu Kα line (λ = 1.54 Å) as the irradiation source.

**Preparation and spectral acquisition of FT-IR samples**

The chromatographic samples were cultured in a drying oven at 70℃ for 10h, and then the solvent was removed to obtain the solid samples of CDs, CDs-Ce6 and *p*-CDs. The prepared solid sample was evenly mixed with potassium bromide at the ratio of 1:100, and the spectrum was collected by infrared spectrometer (Thermo Scientific Nicolet iZ 10) after pressing. The air background is subtracted before the sample is tested.

**Fluorescence quantum yield and fluorescence lifetime**

Fluorescence quantum yields and fluorescence lifetime were measured on the spectrophotometer (FLS1000) equipped with maximum absorption wavelength. The CDs, CDs-Ce6, *p*-CDs, *p*-CDs+RNA-1, *p*-CDs+RNA-2 were diluted from a stock solution in different solvents to achieve an absorbance =0.1 at maximum absorption wavelength nm in solution. The compounds were excited at maximum absorption wavelength in a quartz colorimetric dish at 298 K. Experiments were performed in air and emission spectra were corrected for the spectral sensitivity of the detection system by standard correction curves. Quantum yields and fluorescence lifetime in solution were read out directly through the instrument.

**The CL QY measurement of the CDs, CDs-Ce6, CDs+Ce6 and Ce6**

The CL QYs of the CDs, CDs-Ce6, CDs+Ce6 and Ce6 were measured using lucigenin as a reference with a known QY of 11.3×10^−3^ einsteins mol^–1^ at pH = 13.8 according to our previous reports. With the CL spectra and kinetic curves, the CL QYs were calculated according to the following equations:

$\boldsymbol{\emptyset}_{\boldsymbol{CL}}\boldsymbol{=}\frac{\boldsymbol{Q\times}\boldsymbol{f}_{\boldsymbol{luc}}\boldsymbol{\times}\boldsymbol{f}_{\boldsymbol{photo}}}{\boldsymbol{n}}\boldsymbol{(}\boldsymbol{einsteins}/\boldsymbol{mol}\boldsymbol{)}$ (1)

$\boldsymbol{f}_{\boldsymbol{luc}}\boldsymbol{=}\frac{\boldsymbol{\emptyset}_{\boldsymbol{luc}}\boldsymbol{\times}\boldsymbol{n}_{\boldsymbol{luc}}}{\boldsymbol{Q}_{\boldsymbol{luc}}}$ (2)

$\boldsymbol{f}_{\boldsymbol{p}\boldsymbol{h}\boldsymbol{oto}}\boldsymbol{=}\frac{\boldsymbol{f}\boldsymbol{(}\boldsymbol{\lambda}_{\boldsymbol{s}}\boldsymbol{)}}{\boldsymbol{f}\boldsymbol{(}\boldsymbol{\lambda}_{\boldsymbol{luc}}\boldsymbol{)}}$ (3)

where *Փ* is the CL QY of the CDs, CDs-Ce6, CDs+Ce6 and Ce6; Q is the total light emission obtained by integration of emission intensity under time curves. In the equation, *f*_luc_ is obtained by measuring the emission kinetics of lucigenin reaction performed in standard conditions (λ = 475 nm). *f*_photo_ is obtained from the sensitivity of CL spectrum normalized at the emission wavelength (λ = 475 nm) of the lucigenin standard, *f*(*λ*_luc_), and at the maximal emission wavelength of the CDs, CDs-Ce6, CDs+Ce6 and Ce6, *f*(*λ*_s_). n is the number of moles of lucigenin (*n*_luc_) or the number of moles of CPPO (*n*_cppo_).

**The NMR spectra were acquired through the following procedure**

The chromatographic samples were incubated at 70 °C in a drying oven for 10h. Subsequently, solvent removal was carried out, and some solid powder was dissolved in deuterated chloroform for NMR analysis. ^1^H NMR and ^13^C NMR spectra were acquired in 5 mm NMR tubes at 293 K or 310 K on either Bruker 400/600M AVANCEIII. Chemical shifts were internally referenced to CDCl_3_ (H: 7.26 ppm, C: 77 ppm).

**Ultrafast transient absorption (TA) spectroscopy measurements**

The femtosecond time-resolved transient absorption (fs-TA) measurements were performed a Helios pump probe system (Ultrafast Systems LLC).) in combination with a Ti: sapphire regenerative amplifier (Coherent Legend Elite-1K-HE and an optical parametric amplifier (TOPAS-800-fs). The samples well dispersed in H_2_O and were contained in a 0.7-m quartz cuvette under a continuous magnetic stirring, ensuring that the photoexcited volume of the samples was kept fresh during the fs-TA measurements.

**High-Resolution Mass Spectrometry (HRMS)**

HRMS spectra were obtained by preparing the samples in ultrapure water solution or using NMR samples for infusion into the mass spectrometer (Aglient 7250). The mass spectra were recorded with a scan range of m/z 100–1500 for positive ions or negative ions.

**Photochemical free radicals and single oxygen determination of the *p*-CDs**

In photochemical reactions, electrons and holes generated from photocatalyst reacting with O_2_ or H_2_O to form oxygen species. With (5,5-dimethyl-1-pyrroline N-oxide (DMPO), 2,2,6,6-tetramethylpiperidine-1-oxyl (TEMPO) and 2,2,6,6-tetramethyl-1-piperidine (TEMP) as probes, the content of •O_2_^−^, ^1^O_2_, and •OH was detected by electron paramagnetic resonance (ESR). The main active species including •O_2_^−^, ^1^O_2_, and •OH could be produced by these *p*-CDs under visible light irradiation. 500 mM of DMPO/TEMPO/TEMP aqueous solution was prepared for ESR testing. Then, 2 mL of sample (1 mg mL^–1^) were mixed with 0.5 mL of TEMP solution and the mixtures were conducted with ESR without light treatment.

**Cell culture**

A549 cells were cultured in Roswell Park Memorial Institute (RPMI) 1640 medium (Sigma, Sigma-Aldrich, St. Louis, USA, RNBJ4428) with 10 % fetal bovine serum (FBS, Gemini, 900-108). The cells were maintained in an atmosphere of 5 % CO_2_ at 37 °C. Cells were passaged once every 2-3 days, and logarithmic cells were taken for experiments.

**Ethics statement and Animals**

All animal experimental procedures and protocols were conducted in accordance with guidelines for ethical of laboratory animal welfare of the Zhengzhou University and the First Affiliated Hospital of Zhengzhou University. Six male SPF grade BALB/c-nu/nu mice (4 ~ 5 weeks) were purchased from Beijing Vital River Laboratory Animal Technology and maintained in SPF conditions (24 ± 1 °C, 12 h light/dark cycle) with free access to water and chow for two weeks before animal experiment.

**Supplementary Figures**

**
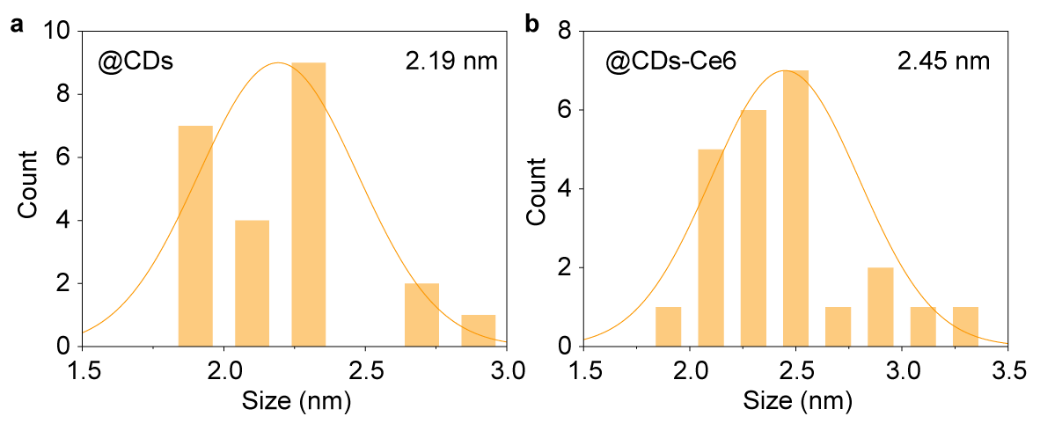
**

**Figure S1.** Size distribution statistics of the CDs and CDs-Ce6.

**
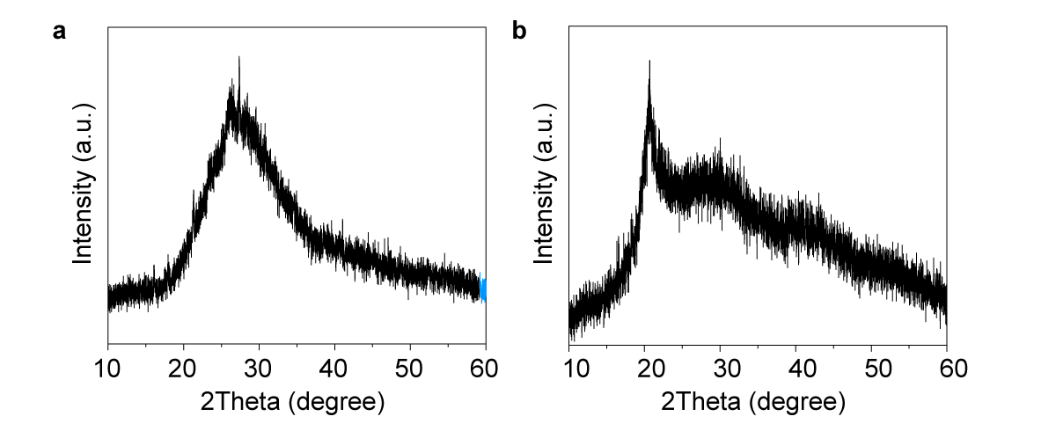
**

**Figure S2.** XRD patterns of the CDs (a) and CDs-Ce6 (b).

**
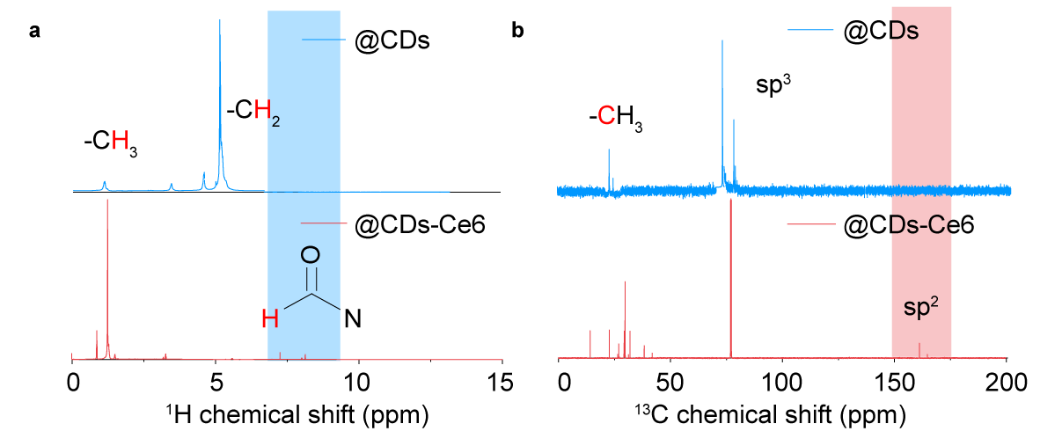
**

**Figure S3.** ^1^H and ^13^C NMR spectra of the CDs (a) and CDs-Ce6 (b).

**
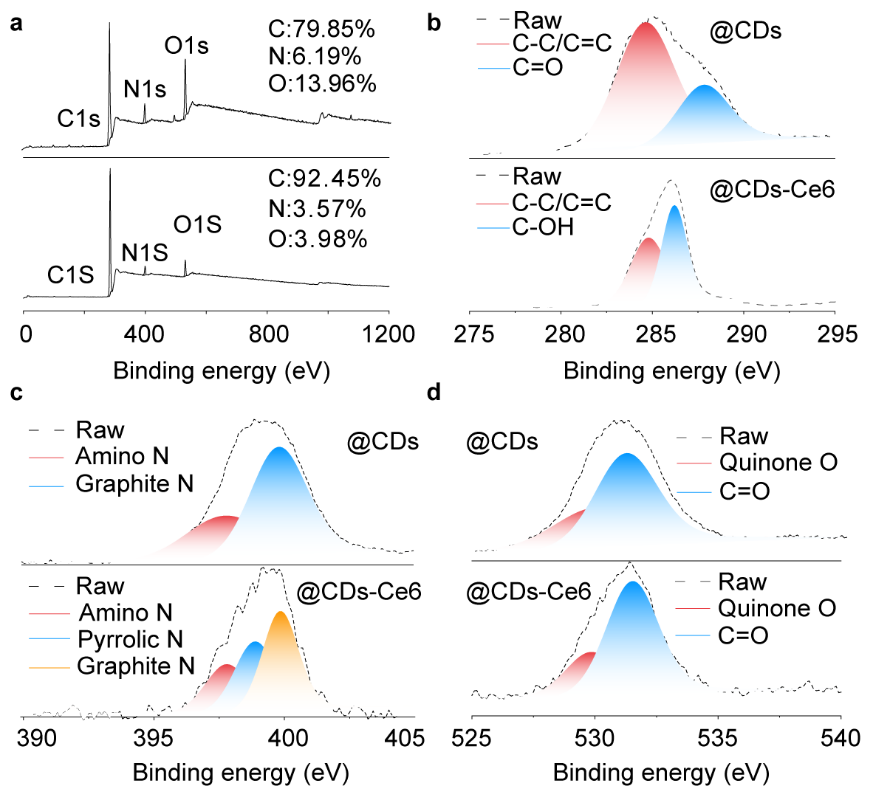
**

**Figure S4.** XPS analysis for the CDs and CDs-Ce6. The XPS full-survey spectra of the CDs and CDs-Ce6 (a), and the XPS spectra C1s (b), N1s (c) and O1s (d) for the CDs and CDs-Ce6.

**
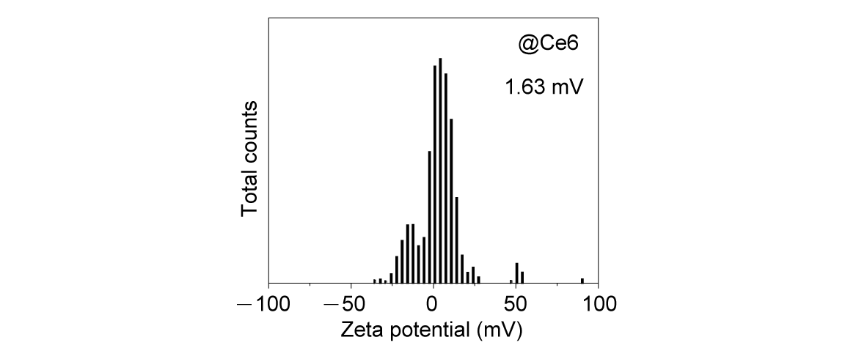
**

**Figure S5.** Zeta potential of the Ce6.

**
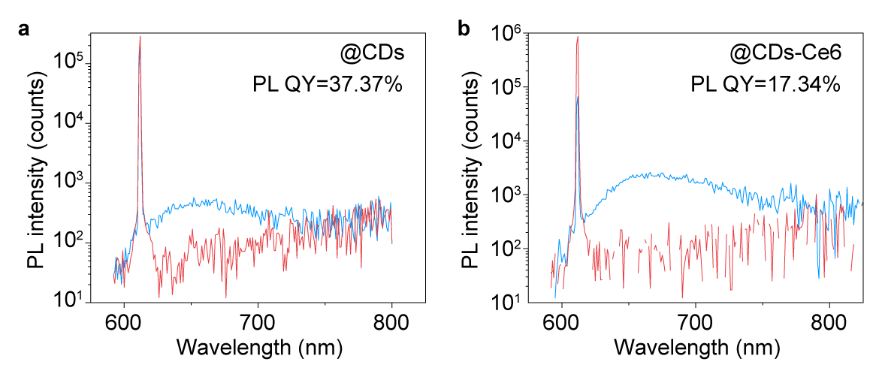
**

**Figure S6.** PL QYs of the CDs and CDs-Ce6. The PL emission spectra with and without the sample for the CDs (a) and CDs-Ce6 (b).

**
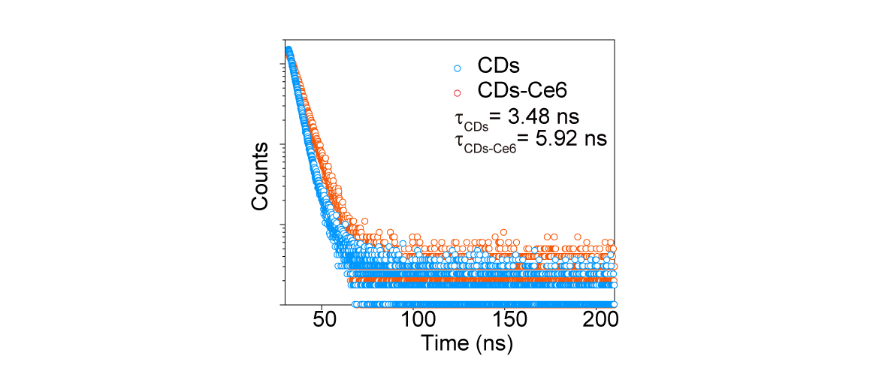
**

**Figure S7.** The fluorescence resolved intensity of CDs and CDs-Ce6.

**
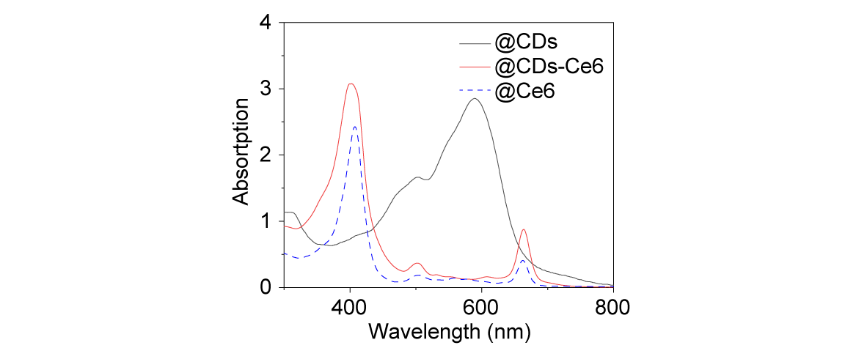
**

**Figure S8.** UV-vis absorption spectra of the CDs, Ce6 and CDs-Ce6.

**
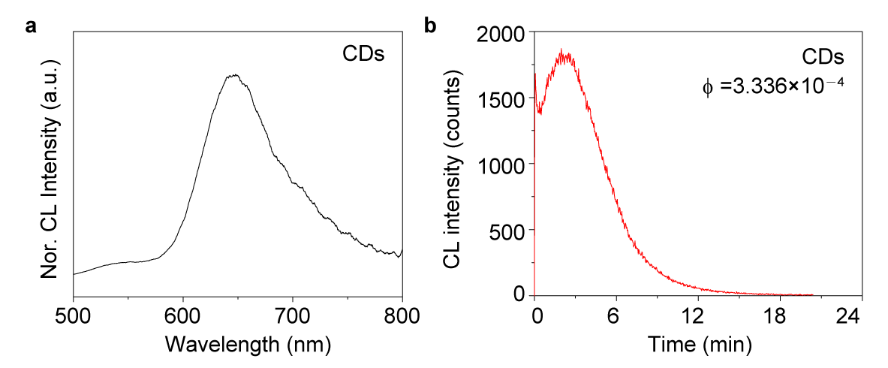
**

**Figure S9.** CL emission spectrum and intensity decay of CDs–CPPO–H_2_O_2_ system. The CL spectrum of CDs–CPPO–H_2_O_2_ (a) and the CL intensity decay curves at 650 nm after adding H_2_O_2_ into the CDs and CPPO solution (b) (EM slit = 20 nm, PMT voltage = 950 V).

**
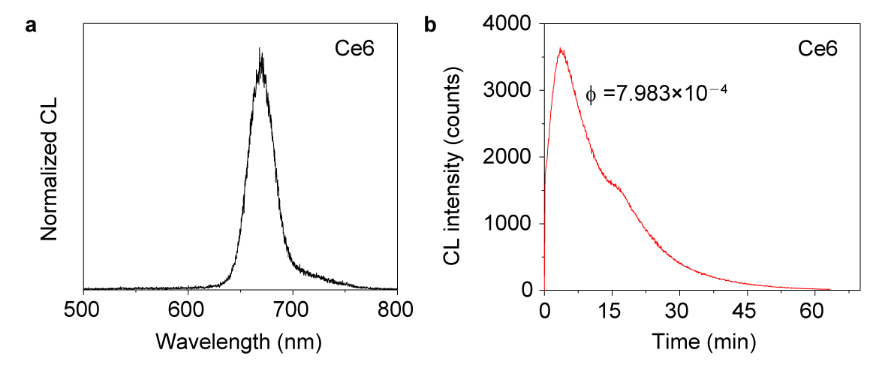
**

**Figure S10.** CL emission spectrum and intensity decay of Ce6–CPPO–H_2_O_2_ system. The CL spectrum of Ce6–CPPO–H_2_O_2_ (a) and the CL intensity decay curves at 670 nm after adding H_2_O_2_ into the Ce6 and CPPO solution (b) (EM slit = 20 nm, PMT voltage = 950 V).

**
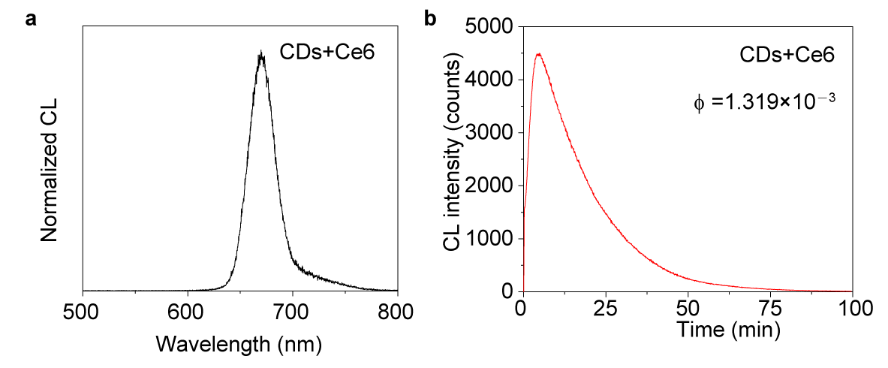
**

**Figure S11.** CL emission spectrum and intensity decay of CDs+Ce6-CPPO-H_2_O_2_ system. The CL spectrum of CDs+Ce6–CPPO–H_2_O_2_ (a) and the CL intensity decay curves at 670 nm after adding H_2_O_2_ into the CDs+Ce6 and CPPO solution (b) (EM slit = 20 nm, PMT voltage = 950 V).

**
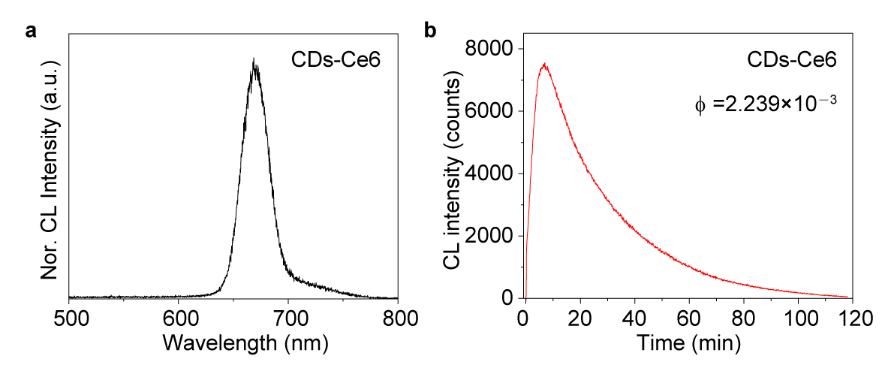
**

**Figure S12.** CL emission spectrum and intensity decay of CDs-Ce6-CPPO-H_2_O_2_ system. The CL spectrum of CDs-Ce6–CPPO–H_2_O_2_ (a) and the CL intensity decay curves at 670 nm after adding H_2_O_2_ into the CDs-Ce6 and CPPO solution (b) (EM slit = 20 nm, PMT voltage = 950 V).


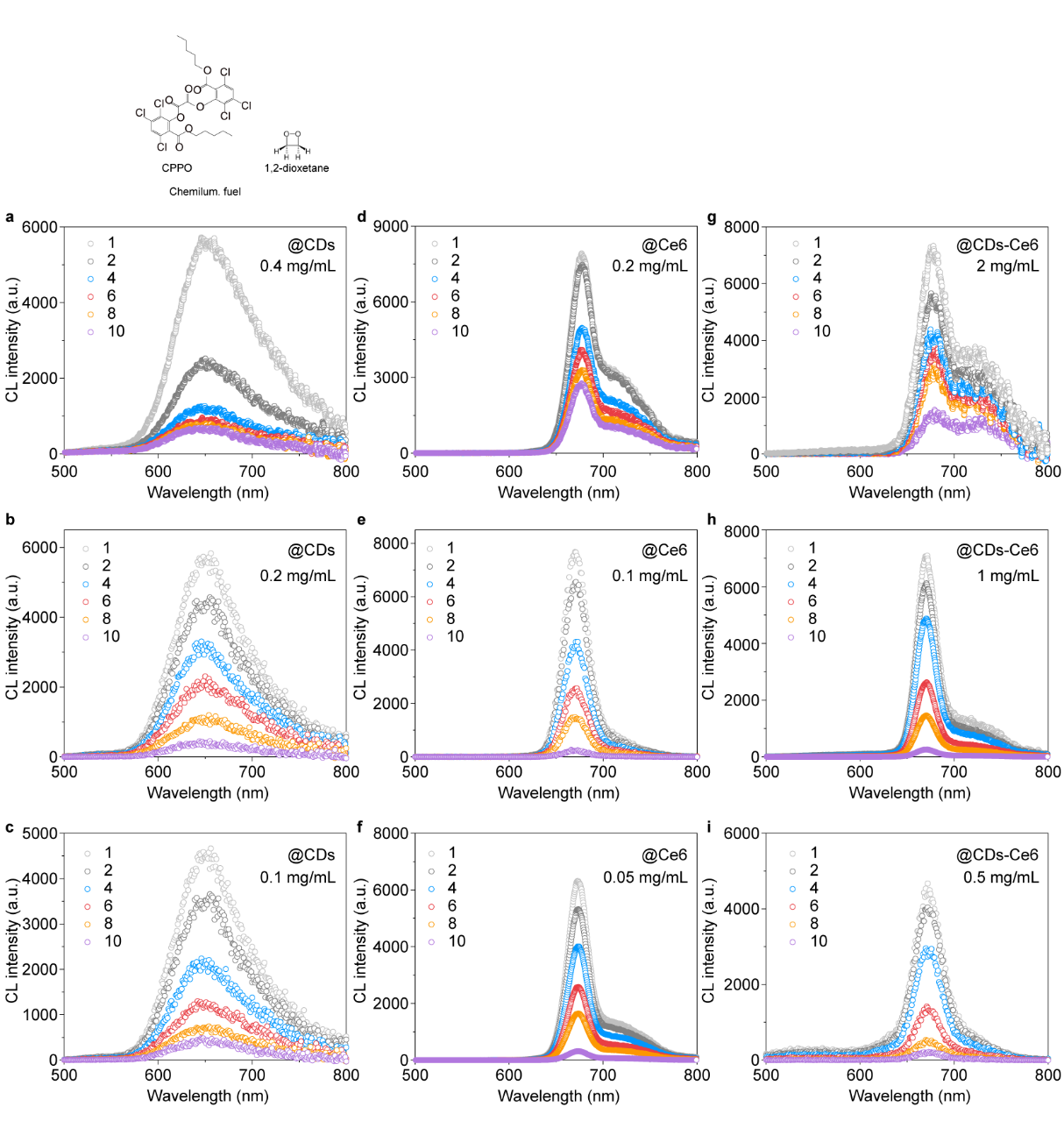


**Figure S13.** The lasting CL emission spectra from the CL reaction with 0.4 mg mL^–1^ (a), 0.2 mg mL^–1^ (b), 0.1 mg mL^–1^ (c) CDs, 0.2 mg mL^–1^ (d), 0.1 mg mL^–1^ (e), 0.05 mg mL^–1^ (f) Ce6, 2 mg mL^–1^ (g), 1 mg mL^–1^ (h), and 0.5 mg mL^–1^ (i) CDs-Ce6 in the solution of CPPO (mg mL^–1^) and H_2_O_2_ (mg mL^–1^) (slit = 10 nm and voltage = 700 V).

**
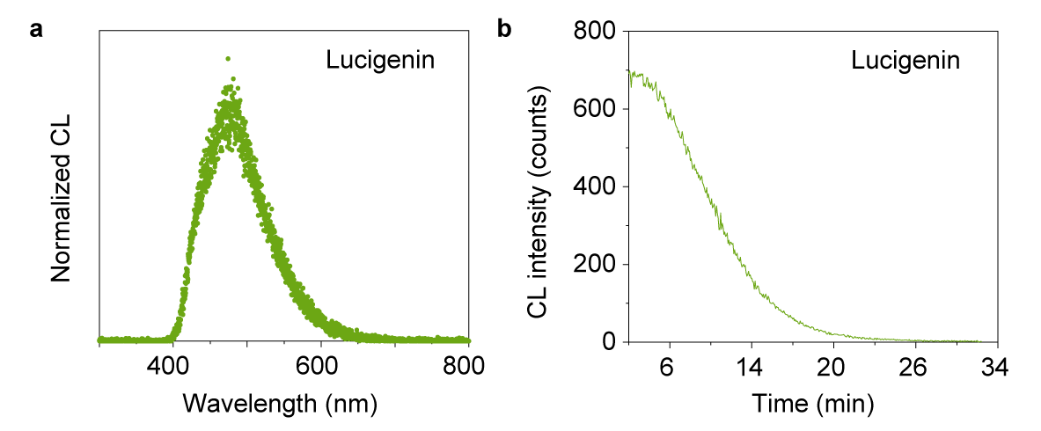
**

**Figure S14.** CL emission spectrum and intensity decay of lucigenin–H_2_O_2_ system. The CL spectrum of lucigenin–H_2_O_2_ (a) and the CL intensity decay curves at 475 nm after adding H_2_O_2_ into the lucigenin solution (b) (EM slit = 20 nm, PMT voltage = 950 V).





**Figure S15.** The TEM image of Ce6@F127.

**
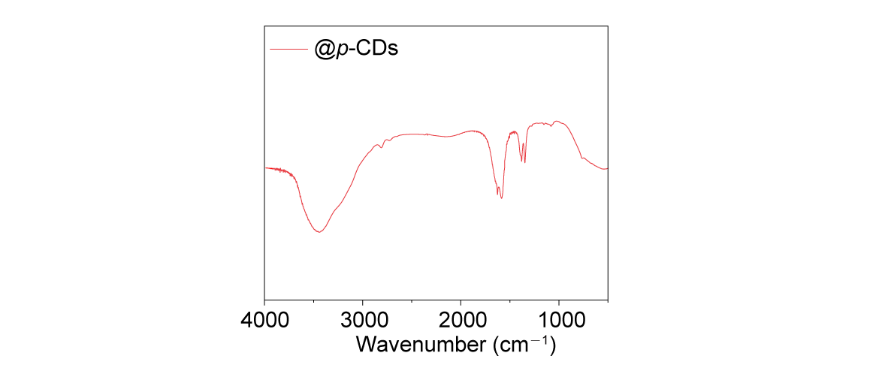
**

**Figure S16.** FT-IR spectrum of the *p*-CDs.

**
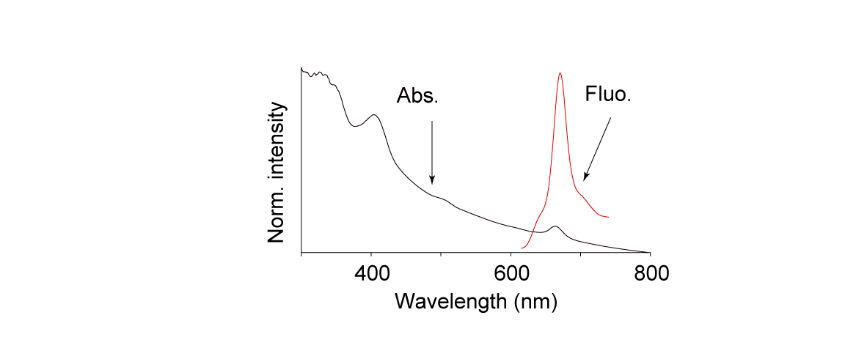
**

**Figure S17.** UV-vis absorption spectra and fluorescence emission spectra of the *p*-CDs.


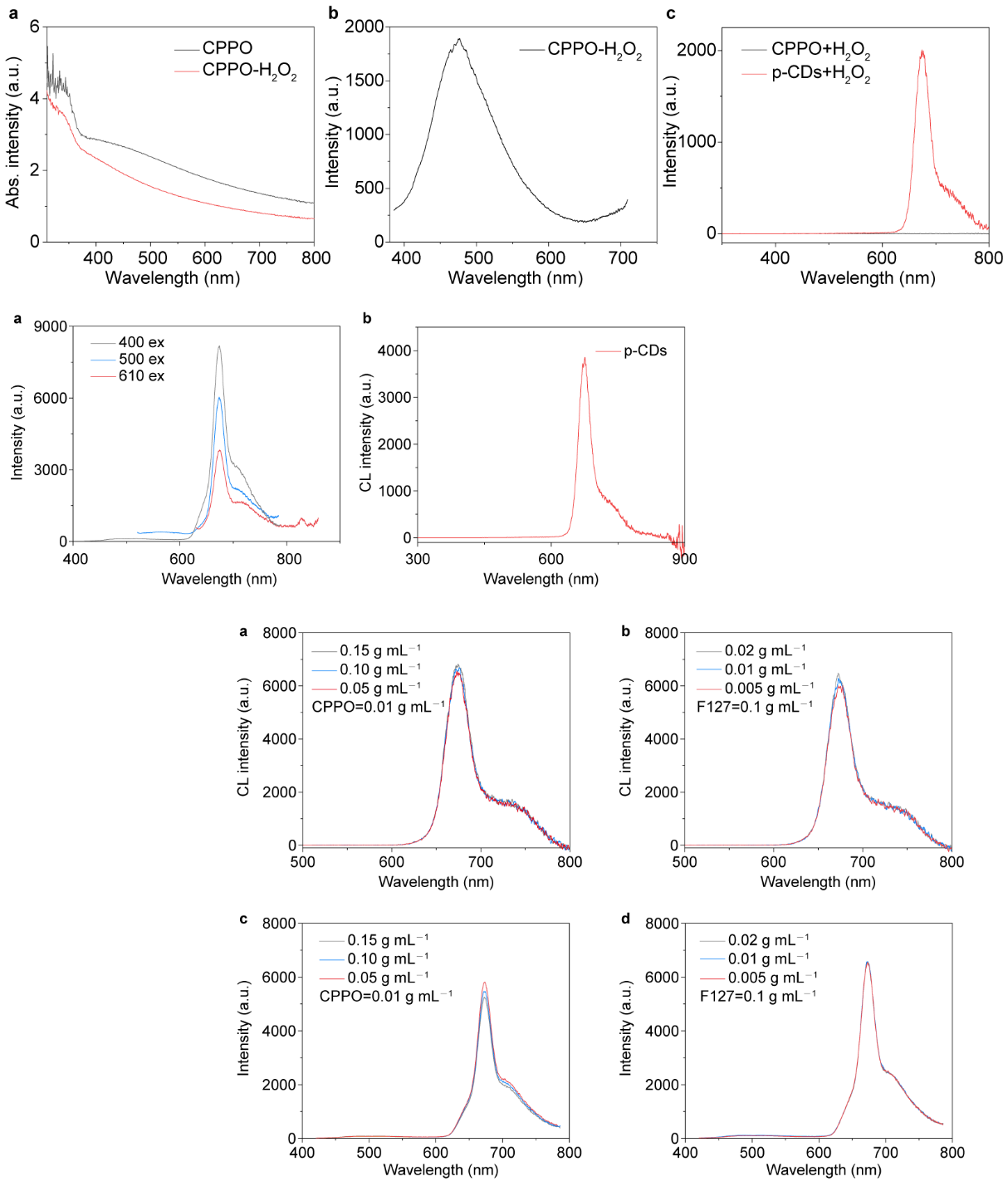


**Figure S18.** a) UV absorption spectra of the CPPO and CPPO-H_2_O_2_ solution. b) Fluorescence spectrum of the CPPO-H_2_O_2_ under 365 nm excitation. c) CL spectrum of the CPPO-H_2_O_2_ and *p*-CDs+H_2_O_2_ solution (slit = 10 nm and voltage = 700 V).


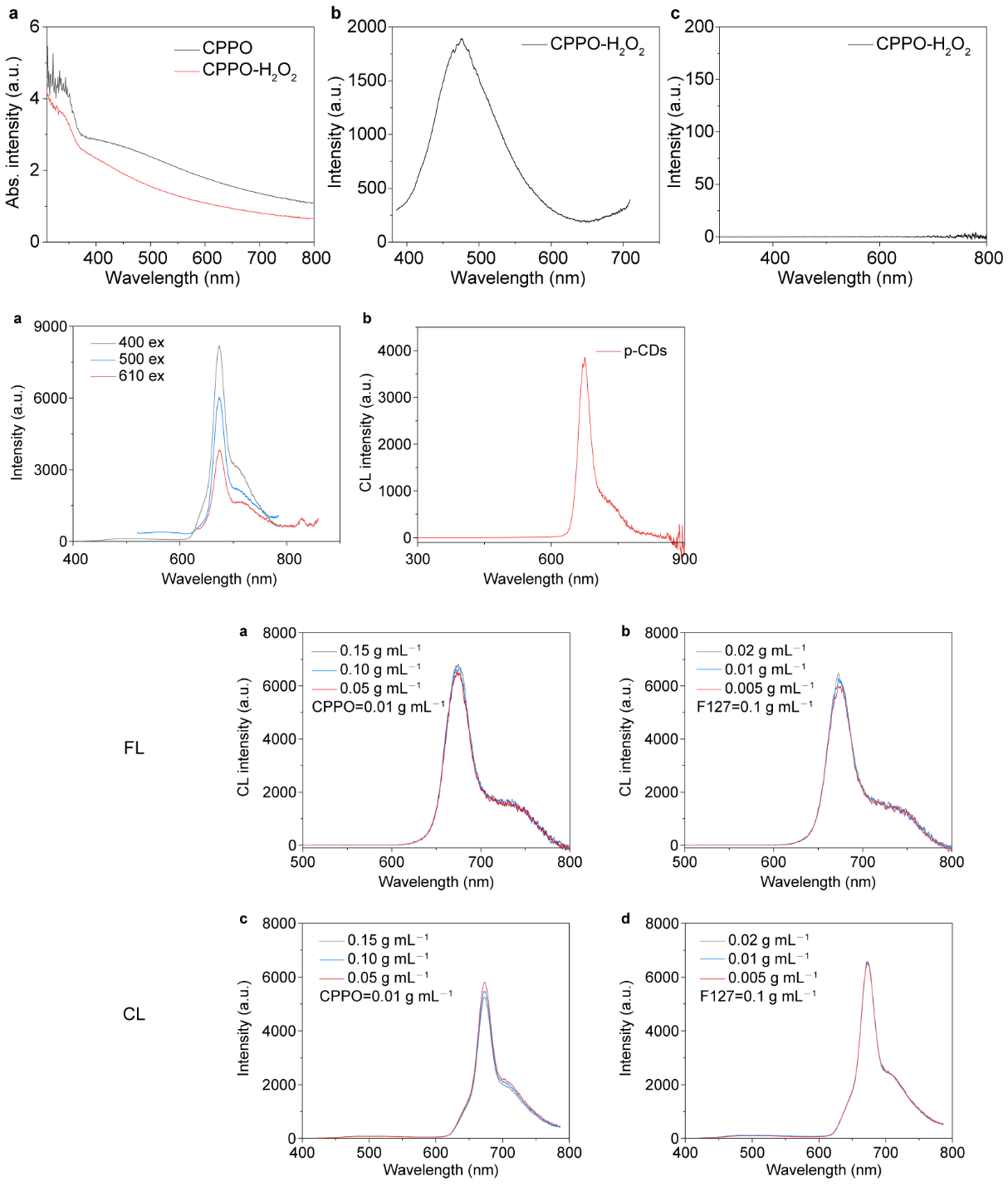


**Figure S19.** CL spectra (a), PL spectra (c) of the *p*-CDs prepared with the CPPO (g mL^–1^), CDs-Ce6 (g mL^–1^) and F127 (g mL^–1^). The CL spectra (b) and PL spectra (d) of the *p*-CDs prepared the CPPO (g mL^–1^), CDs-Ce6 (g mL^–1^) and F127 (g mL^–1^).


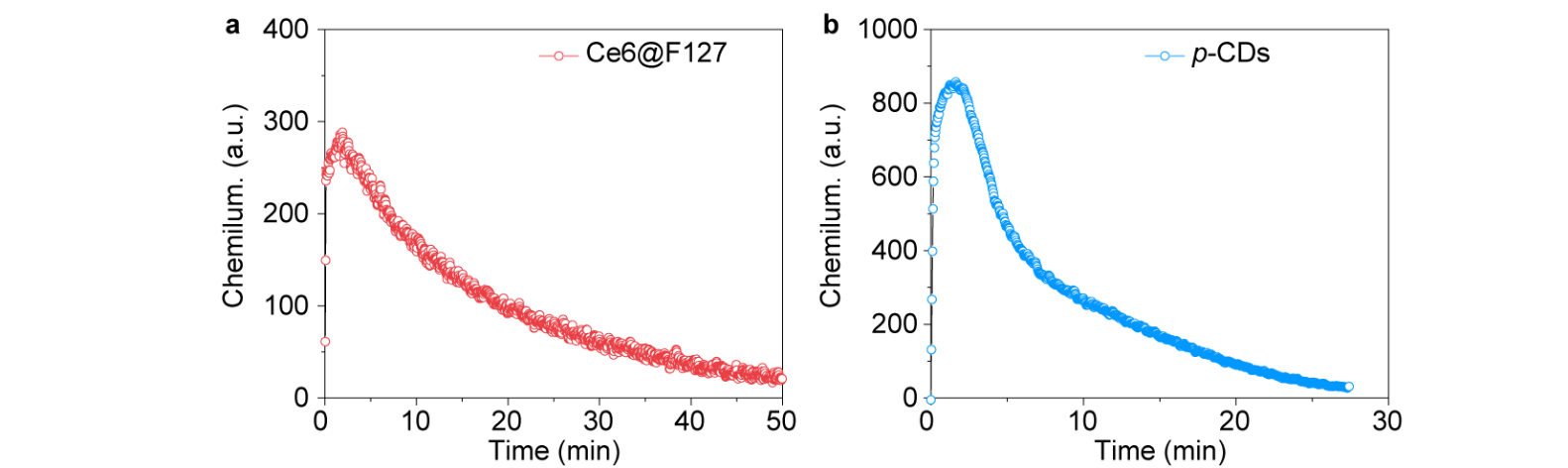


**Figure S20.** The CL decay of Ce6@127 (a) and *p*-CDs (b).


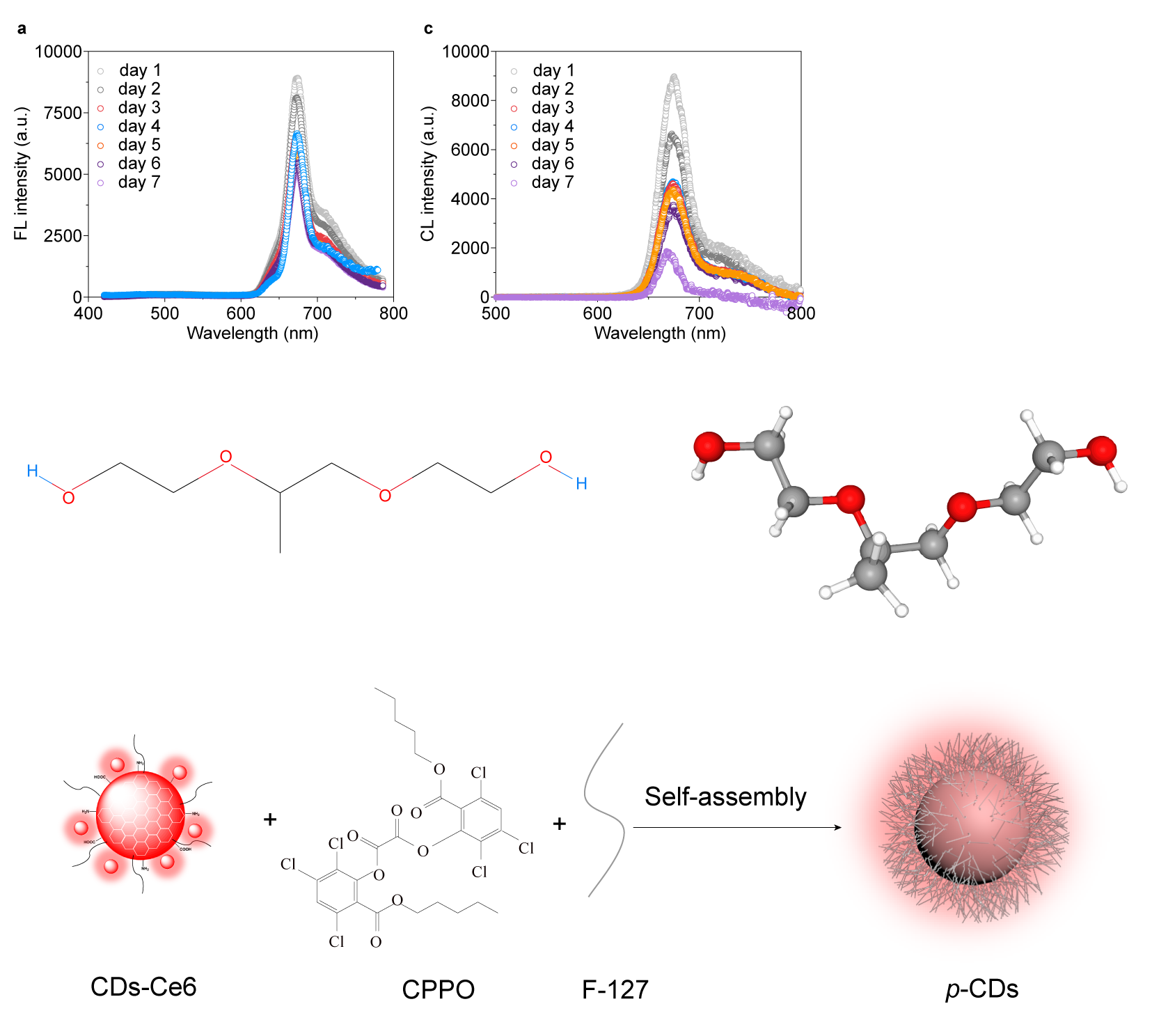


**Figure S21.** Fluorescence characteristics (a) and CL characteristics (b) of *p*-CD during 7 days under shaded refrigerated and airtight conditions.


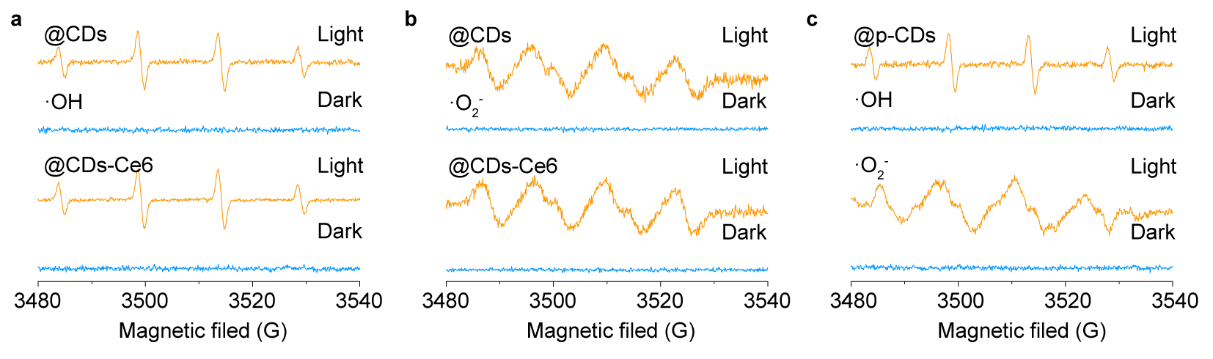


**Figure S22.** ESR spectra of the CDs, CDs-Ce6 and *p*-CDs under light irradiation or dark for the hydroxyl radicals (•OH, •O_2_^–^).


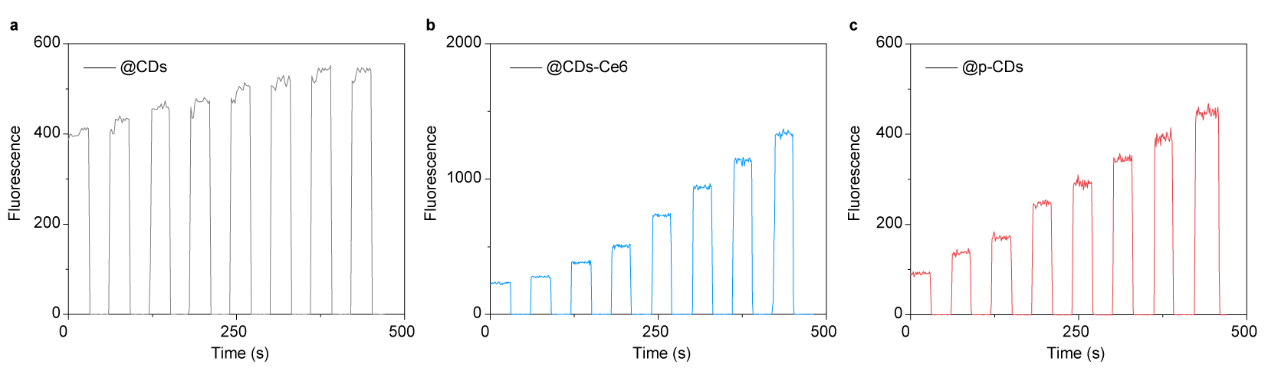


**Figure S23.** The fluorescence intensity of of SOSG for the ^1^O_2_ production after CDs+SOSG (a), CDs-Ce6+SOSG (b) and *p*-CDs+SOSG (c) aqueous solution treated with the cycle of illumination (30 s) under dark.

**
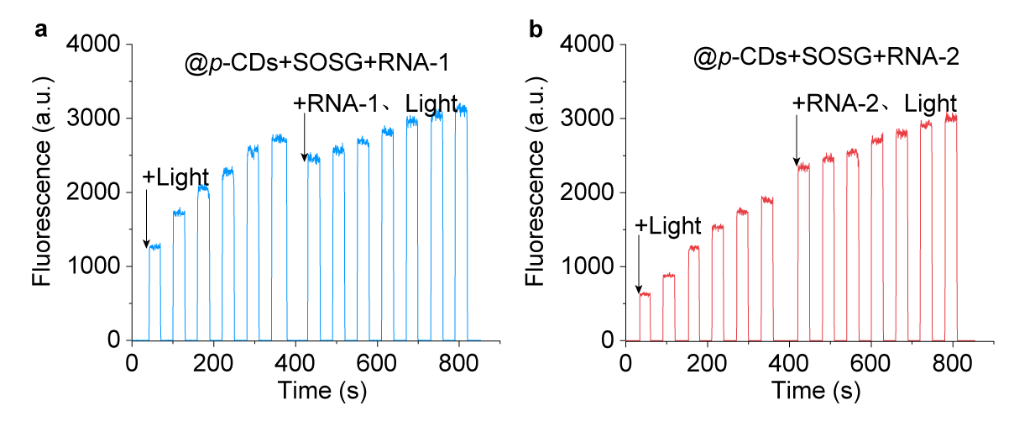
**

**Figure S24.** The fluorescence intensities of SOSG for the ^1^O_2_ production after the *p*-CDs+SOSG+RNA-1 (a) and *p*-CDs+SOSG+RNA-2 (b) aqueous solutions treated with the cycle of illumination (30 s) under dark.

**
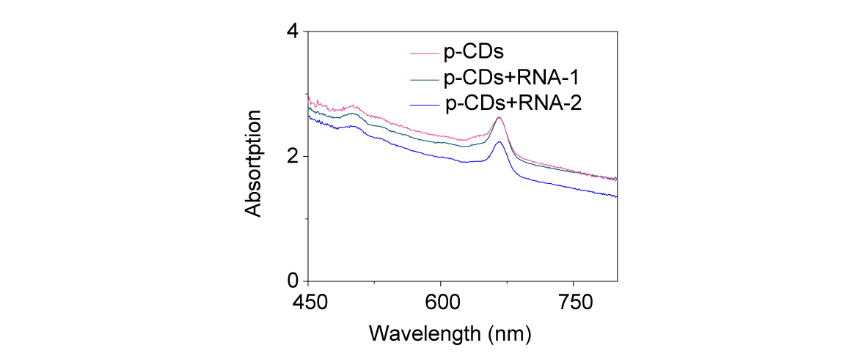
**

**Figure S25.** UV-vis absorption of the *p*-CDs, *p*-CDs+RNA-1 and *p*-CDs+RNA-2 (C_RNA_: 4 nmol/mL).


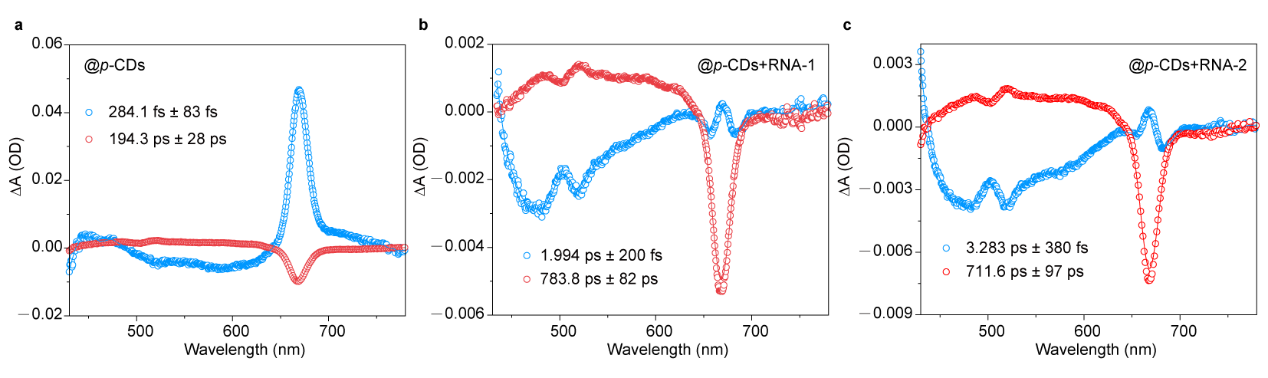


**Figure S26.** The influence of RNA on the TA of *p*-CDs. Nanosecond time-resolved TA spectra of the *p*-CDs (a), *p*-CDs+RNA-1 (b) and *p*-CDs+RNA-2 (c) (C_RNA_: 4 nmol/mL).


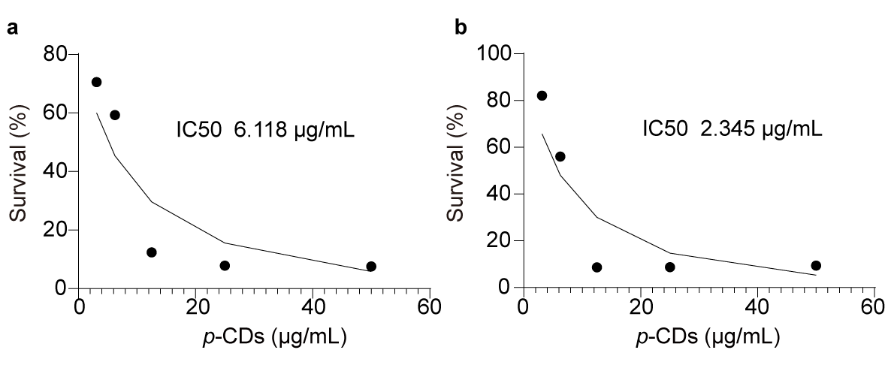


**Figure S27.** Relative viabilities of with the different concentrations of *p*-CDs.

**Table S1.** The comparison between the proposed CDs-Ce6-based system and lanthanide-doped nanoparticle.

|  | Wavelength | Lifetime | Ref |
| --- | --- | --- | --- |
| NaYF4 : Ln^3+^ @NaYF4(Ln=Tb, Er, Dy, Ho, Tb@Eu, Nd) | 480-1064 | 30 min | *Light-Sci. Appl. 2021, 10, 1-10* |
| Zn_3_Ga_2_GeO_8_ : Cr^3+^,Yb^3+^,Er^3+^ | 706 | 30 min | *Adv. Funct. Mater. 2020, 30, 1-9* |
| LaGaO_3_ : Sb^3+^,Cr^3+^ | 750 | 150 h | *Chem. Eng. J. 2021, 404, 1-32* |
| NaLuF_4_ : Gd^3+^,Tm^3+^ | 800 | 58 min | *Ceram. Int. 2023, 49, 39664-39670* |
| MgGeO_3_: Mn^2+^, Yb^3+^, Li^+^ | 1000 | 3 h | *Nanoscale 2020, 12, 14037-14046* |
| MgGeO_3_: Yb^2+^, Yb^3+^ | 1019 | 30 min | *ACS Appl. Mater. Interfaces 2021, 13, 16166-16172* |
| Ln-NaY(Gd)F₄ (LN=Nd^3+^, Ho^3+^, Tm^3+^, Er^3+^) | 1525 | 12 h | *Nat. Nanotechnol. 2021, 16, 1011-1018* |
